# Sexual Contact Patterns in High-Income Countries—A Comparative Analysis Using Data From Germany, the United Kingdom, and the United States

**DOI:** 10.3389/fepid.2022.858789

**Published:** 2022-05-04

**Authors:** Damilola Victoria Tomori, Johannes Horn, Nicole Rübsamen, Sven Kleine Bardenhorst, Christoph Kröger, Veronika K. Jaeger, André Karch, Rafael Mikolajczyk

**Affiliations:** ^1^Institute of Epidemiology and Social Medicine, University of Münster, Münster, Germany; ^2^Institute for Medical Epidemiology, Biometrics and Informatics, Interdisciplinary Center for Health Sciences, Martin Luther University Halle-Wittenberg, Halle, Germany; ^3^Institute of Psychology, University of Hildesheim, Hildesheim, Germany

**Keywords:** contact patterns, sexual behavior, mathematical model, sexually transmitted infections, population-based survey

## Abstract

Sexual contact patterns determine the spread of sexually transmitted infections and are a central input parameter for mathematical models in this field. We evaluated the importance of country-specific sexual contact pattern parametrization for high-income countries with similar cultural backgrounds by comparing data from two independent studies (HaBIDS and SBG) in Germany, a country without systematic sexual contact pattern data, with data from the National Survey of Sexual Attitudes and Lifestyles (Natsal) in the UK, and the National Survey of Family Growth (NSFG) in the US, the two longest running sexual contact studies in high-income countries. We investigated differences in the distribution of the reported number of opposite-sex partners, same-sex partners and both-sex partners using weighted negative binomial regression adjusted for age and sex (as well as stratified by age). In our analyses, UK and US participants reported a substantially higher number of lifetime opposite-sex sexual partners compared to both German studies. The difference in lifetime partners was caused by a higher proportion of individuals with many partners in the young age group (<24 years) in the UK and the US. Partner acquisition in older age groups was similar. The number of same-sex partners was similar across countries, while there was heterogeneity in the reported experience with partners from both sexes, consistent with the differences observed for opposite-sex sexual partners. These patterns can lead to substantially different dynamics of sexually transmitted infections across ages, and have strong impact on the results of modeling studies.

## Introduction

Sexually Transmitted Infections (STIs) are caused by over 30 different bacteria, viruses and parasites (1), and are a major threat to global public health, with more than 350 million curable STI cases (1) diagnosed each year. Behavioral factors associated with the number of sexual partners, the concurrency of sexual partnerships, and the use of preventive barrier devices like condoms are crucial for the transmission dynamics of STIs (2). The so-called sexual contact patterns are subject to change, and can depend on cultural habits, religious beliefs, and societal norms (2, 3).

Several studies have assessed the relationship between sexual contact patterns and various sexually transmitted infections (2, 4–6), and were e.g., crucial in the understanding of the role of concurrent partnerships for the heterosexual spread of HIV in Sub-Saharan Africa (4). Information about sexual contact patterns is important for the parametrization of mathematical models studying STI transmission, which are used to guide prevention efforts. However, there is little systematic evidence for population-based estimates on the frequency of various types of sexual contact patterns in many geographic and cultural settings. The scarcity of research is often attributed to the fact that surveys on sexual contacts are generally sensitive and privy topics for discussion.

There are few large-scale projects like Natsal [National Survey of Sexual Attitudes and Lifestyles, Natsal (7)] in the United Kingdom and NSFG in the United States [National Survey of Family Growth (8, 9)], which provide long-term, high-quality data for selected high-income countries. Sexual contact patterns reported in these two projects are commonly used for modeling STI transmission in countries without systematic data collection, like, e.g., Germany. The main objective of this study is to provide population-level estimates for sexual contact patterns in Germany, and to compare them to data obtained from Natsal and NSFG for the UK and US.

## Materials and Methods

### Data Source, Setting, and Study Population

This study uses data from the Hygiene and Behavior Infectious Diseases Survey (HaBIDS) panel which included individuals from four counties in the Federal State of Lower Saxony, Germany (Braunschweig, Salzgitter, Vechta, and Wolfenbüttel) and was run between 2014 and 2017 (10). HaBIDS was designed to study knowledge, attitudes, and practice toward infectious diseases from various perspectives (10–12). Potential participants aged between 15 and 69 years were selected from the population registries of the respective counties by proportional stratified random sampling, and were invited by mail to participate initially in a mixed method (paper-pencil and online) and later on in an online only panel. A series of questionnaires regarding behavior in the context of infectious diseases including sexual contact behavior was sent to all participants. Data on sexual contacts were collected using paper-based (in July 2014) and online-based questionnaires (in June 2015). Further details on the HaBIDS study and its methodology can be found in Rübsamen et al. (10). At the initial stage, the HaBIDS panel consisted of 2,379 individuals from a registration office sample of 26,895 (9% participation rate). At the time of data collection on sexual contact behavior, 123 individuals had already resigned from HaBIDS, 740 individuals did not send back the questionnaire and 318 individuals either found the questionnaire too personal, returned an empty questionnaire, or gave no information on the number of sexual partners, so that 1,198 participants were left for this analysis.

In the questionnaire, participants were asked if they had a sexual contact ever in their life, at which age they had their first sexual contact, how many sexual partners (opposite- and same-sex) they had during their entire life, during the last 12 months and how many new sexual partners they had during the last 12 months. Moreover, participants were asked to report the age and sex of their last three sexual partners, which we refer to as last/current partner, penultimate partner, and pre-penultimate partner. A translated English version of the questionnaire can be found in the Supplementary Material. HaBIDS was approved by the Ethics Committee of Hannover Medical School (No. 2021-2013), and by the Federal Commissioner for Data Protection and Freedom of Information in Germany. All participants provided written informed consent prior to study entry. Informed consent was obtained from a parent or guardian on behalf of any participants aged 18 or below. The HaBIDS study was performed in accordance with relevant guidelines and regulations.

We compared HaBIDS data to sexual contact patterns from a recent nationwide survey on sexual behavior in Germany (SBG) (13) (Table 1). Sexual contact patterns (in contrast to other sexual behavior data) from this survey were not published before but were provided to us by the SBG authors. SBG participants were aged between 14 and 99 years at data collection in 2016. The included households were identified by subdividing the regional areas using the random route method; potential participants were selected using the kish selection grid (13).

**Table 1:** Basic information on HaBIDS, SBG, Natsal, and NSFG.

**Study**	**Country**	***N* (participants)**	**Age range (years)**
HaBIDS	Germany	1,198	15–69
SBG	Germany	2,096	14–99
Natsal	UK	15,118	16–74
NSFG	USA	22,457	15–44

We also compared HaBIDS and SBG to two long-term projects from the UK (Natsal) (7) and the US (NSFG) (8, 9) (Table 1). We chose Natsal and NSFG as comparison studies as they are the longest-running platforms for sexual contact studies in high-income countries and provide robust data for sexual contact patterns in the respective countries. For those studies with more than one survey wave, we chose the wave closest to the date of the HaBIDS survey because this survey was only conducted once and the potential effects of secular trends needed to be minimized. This means for example, that we decided to use data from Natsal wave 3 which were collected between 2010 and 2012 as wave's 3 data were sampled closest in time to the HaBIDS data. Natsal used a multistage, clustered and stratified probability sample design and enrolled participants between the ages of 16 and 74 years (7). Data used in this publication from the NSFG study were sampled in the 2006–2010 survey. NSFG included participants aged 15–44 years based on an independent, national probability sampling (8, 14). For all studies, individual anonymized primary data were provided and used for further analyses.

Sexual contact patterns were compared between the four studies; the questions to collect these data were similarly phrased across the studies. Data on the number of lifetime sexual partners were available in all four studies. Data on the number of sexual partners in the last 12 months were available for HaBIDS, Natsal, and NSFG; data on the number of new partners in the last 12 months were available for HaBIDS and Natsal. No data were available for NSFG participants above the age of 44 years.

Participants who reported at least one opposite-sex sexual partner and at least one same-sex sexual partner are referred to as participants with “both-sex experience”.

### Data Analysis

We used data from the 2011 census [(15)—*Bevölkerungs- und Wohnungszählung*, 2011] in Germany to apply survey weights to the HaBIDS and SBG study population based on age and sex of the participants to represent the key characteristics of the German population. Natsal participants were weighed according to 2011 census data for Great Britain by age, sex and government office region (7), while sampling weights in NSFG were applied in a similar way to compensate for oversampling, non-response and non-coverage (8). Full details of the weighing methods for Natsal and NSFG can be found elsewhere (7, 8, 16).

Absolute and relative frequencies stratified by weighing factors and weighted medians/means were used for descriptive analyses. Differences in the distribution of the reported number of opposite-sex partners, same-sex partners and both-sex partners were investigated using weighted negative binomial regression adjusted for age and sex (as well as stratified by age). The log-likelihood function for the weighted negative binomial regression was,


$$\begin{array}{l} \;\Lambda \;=\overset{n}{\underset{k=1}{\sum }}\omega_{k}\left(ln\;\left(P\left(Y=\;y_{k}|\;X_{k}\right)\;\right)\right)\; \end{array}$$

where ω_*k*_ is the weighted observation for each κ, *y*_*k*_ is the number of observed sexual contacts, **X**_**k**_ is the vector of covariates or independent variables, and *P* is the probability density function of the discrete negative binomial regression given as:


$$\begin{array}{l} P\left(Y=\;y_{k}|\;X_{k}\right)\\ =\left(\frac{\Gamma \;\left(y_{k}\;+\;\frac{1}{\varphi }\right)}{\Gamma \;\left(\frac{1}{\varphi }\right)\;\Gamma \;\left(y_{k}+1\right)}\right)\;{\left(\frac{1}{1\;+\;\varphi \mu }\right)}^{\frac{1}{\varphi }}{\left(\frac{\varphi \mu }{1\;+\;\varphi \mu }\right)}^{y_{k}}\\ =\;\left(\frac{y_{k}\;+\;\varphi \;-\;1\;}{\varphi \;-\;1}\right)\;{\left(\frac{1}{1\;+\;\varphi \mu }\right)}^{\frac{1}{\varphi }}{\left(\frac{\varphi \mu }{1\;+\;\varphi \mu }\right)}^{y_{k}}\; \end{array}$$

where μ is an invertible log-link function expressed as exp (**X**_**k**_ **β**); **β** is the vector of regression coefficients and φ is the dispersion parameter. Since participants in NSFG are all below 45 years of age, we performed two separate models for each outcome of interest, one across the entire age range including Natsal, HaBIDS and SBG, as well as one excluding participants aged 45 years and above based on Natsal, HaBIDS, SBG and NSFG. Effect estimates for NSFG are taken from the latter model, while effect estimates for the other three surveys are taken from the former model. Both are reported in **Table 3**.

Local regression methods were used to visualize the relationship between the number of sexual partners and the corresponding age of participants in a smoothed way. All analyses were performed using R-3.6.1 (17) with the packages ggplot2 (18), survey (19), and MASS (20).

## Results

### Characteristics of the Samples

Of the 1,198 HaBIDS participants included in this study, 748 were female (62%) and 450 were male (38%). The median age was 44 years [interquartile range (IQR) 42–45 years]; 36 participants (3%) were below the age of 20 years, and 213 participants (18%) were over 60 years old. SBG participants had a median age of 46 years (IQR 44–47), while it was 43 years (IQR 42–43) for Natsal participants and 29 years (IQR 29–29) for NSFG participants. Further information on the study populations can be found in Table 1.

### Current Partnership and Age at Sexual Debut

**Figure 1:**
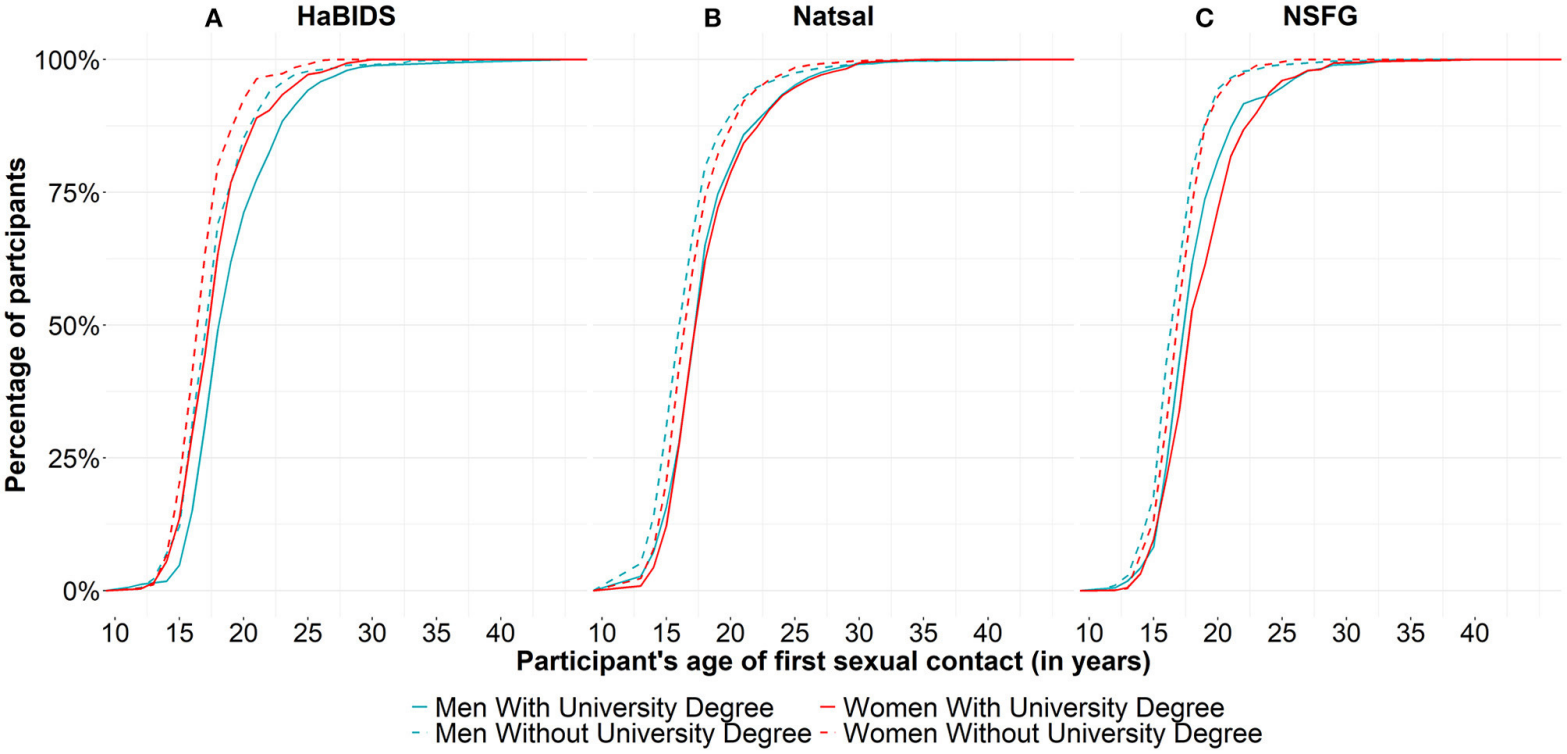
Participants' age of their first sexual contact stratified by education and sex between studies (visualized as a cumulative percentage) for **(A)** HaBIDS, **(B)** Natsal, and **(C)** NSFG. This information was only available for about 10% of NSFG participants.

There were no relevant differences in sexual contact patterns between HaBIDS participants using a paper-based questionnaire and those using an online questionnaire when taking age, sex, and education into account (Supplementary Table 1). At the time of data collection, 83% of the participants had a sexual partner and 60% of those thought that this sexual relationship will also continue for at least 3 months. Thirty participants (2.5%; 20 men, 10 women; median age 18, IQR 17–26) reported that they have never had a sexual contact in their lifetime. Men were older at their sexual debut than women in HaBIDS (median ages 18 vs. 17 years; Table 2; Figure 1) as well as in the other studies.

**Table 2:** Demographic and sexual behavior of the HaBIDS participants (IQR; interquartile range).

	**Men (*****N*** **=** **450)**		**Women (*****N*** **=** **748)**		**Total (*****N*****=** **1,198)**	
	***N***	**Median [IQR]**	***N***	**Median [IQR]**	***N***	**Median [IQR]**
Age of participants (in years)	449	43 [42–45]	748	44 [42–45]	1,197	44 [42–45]
Age of participants sexual debut (in years)	417	18 [18–18]	726	17 [17–17]	1,143	18 [17–18]
	***N***	**%**	***N***	**%**	***N***	**%**
**Marital status**						
Married	263	60	445	61	708	61
Single	138	32	194	27	332	28
Divorced	32	7	68	9	100	8
Widowed	5	1	25	3	30	3
**Highest completed educational level**						
University degree	213	48	274	37	487	42
University entrance qualification	123	28	195	27	318	27
Lower secondary education or apprenticeship	92	21	253	35	345	29
Still student	11	3	10	1	21	2
**Do you currently have a sexual partner?**						
Yes	361	85	594	82	955	83
No	64	15	134	18	198	17
**Do you think this sexual relationship will continue in 3 months?**						
Yes	245	97	328	99	573	97
No	3	1	0	0	3	1
I don't know	6	2	3	1	9	2
**Did your partner have sexual intercourse with other people in the last 12 months?**						
Yes (I know that exactly)	12	3	20	3	32	3
Yes (I suppose, but I'm not sure)	6	2	11	2	17	2
Yes	9	2	15	2	24	2
No	323	80	580	85	903	83
I do not know	54	13	55	8	109	10
**Do you know how many sexual partners your partner had in his/her life in total excluding yourself?**						
Yes, I can give an estimate	139	36	258	40	397	39
I do not know	245	64	388	60	633	61
**How often did your partner use condoms in the past partnerships in his/her life?**						
Always (every intercourse)	28	7	41	6	69	7
Sometimes	54	14	160	25	214	21
Never	30	8	50	8	80	8
I do not know	268	71	396	61	664	64
**In the last 12 months, how often did you use condoms with your last or current partners?**						
Always (every intercourse)	46	12	71	12	117	12
Sometimes	55	15	93	15	148	15
Never	271	73	451	73	722	73
**In the last 12 months, how often did you use condoms with your penultimate partners?**						
Always (every intercourse)	16	30	24	33	40	31
Sometimes	8	15	17	23	25	20
Never	30	55	32	44	62	49
**In the last 12 months, how often did you use condoms with your pre—penultimate partners?**						
Always (every intercourse)	14	36	11	34	25	35
Sometimes	8	20	5	16	13	18
Never	17	44	16	50	33	47

**Figure 2:**
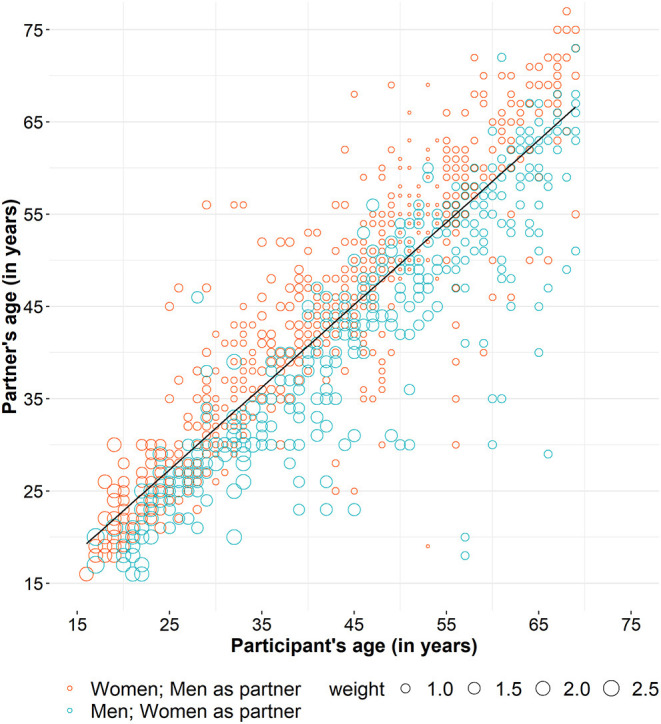
Participants' age and the age of their current partners in HaBIDS.

Participants with a University qualification or University degree were generally older at sexual debut when compared to those without a University qualification (Figure 1). Women reported more often a sexual relationship with older opposite sex partners while men reported frequently to be engaged in sexual relationships with younger opposite sex partners (Figure 2).

### Opposite-Sex Partners

#### Lifetime Opposite-Sex Partners

**Figure 3:**
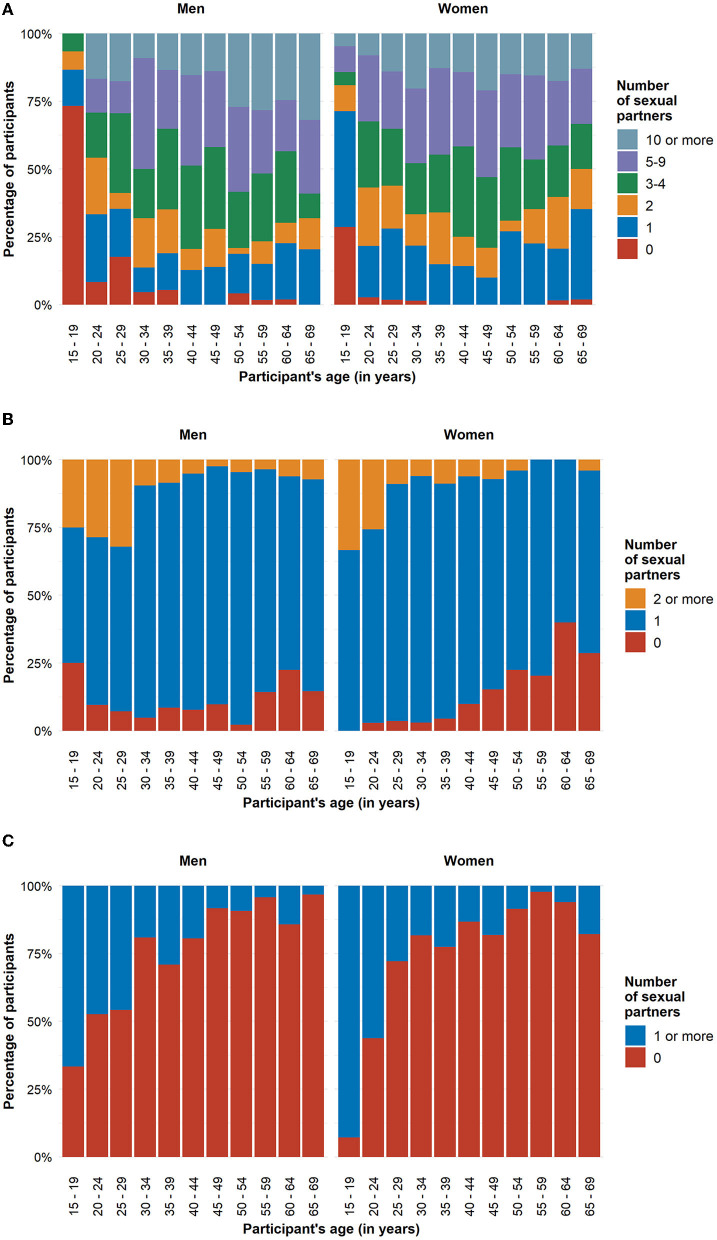
Number of opposite-sex sexual partners in HaBIDS participants stratified by sex **(A)** in the participants' entire life, **(B)** in the last 12 months (based on participants who had at least one lifetime opposite-sex sexual partner), and **(C)** new opposite-sex sexual partners in the last 12 months (based on participants who had at least one opposite-sex sexual partner in the last 12 months).

In HaBIDS, men and women had a median of 3 (IQR 3–4) and 4 (IQR 3–4) lifetime opposite-sex sexual partners respectively. In the group of 15–19 years old participants, 17 participants (46%) reported to have never had an opposite-sex sexual partner; this proportion decreased strongly for older age groups. Forty-three percent of women and 13% of men reported to have had just a single partner in their entire life. Men had lower numbers of lifetime opposite-sex sexual partners than women in the younger age groups, and higher numbers in older age groups (Figure 3A).

**Figure 4:**
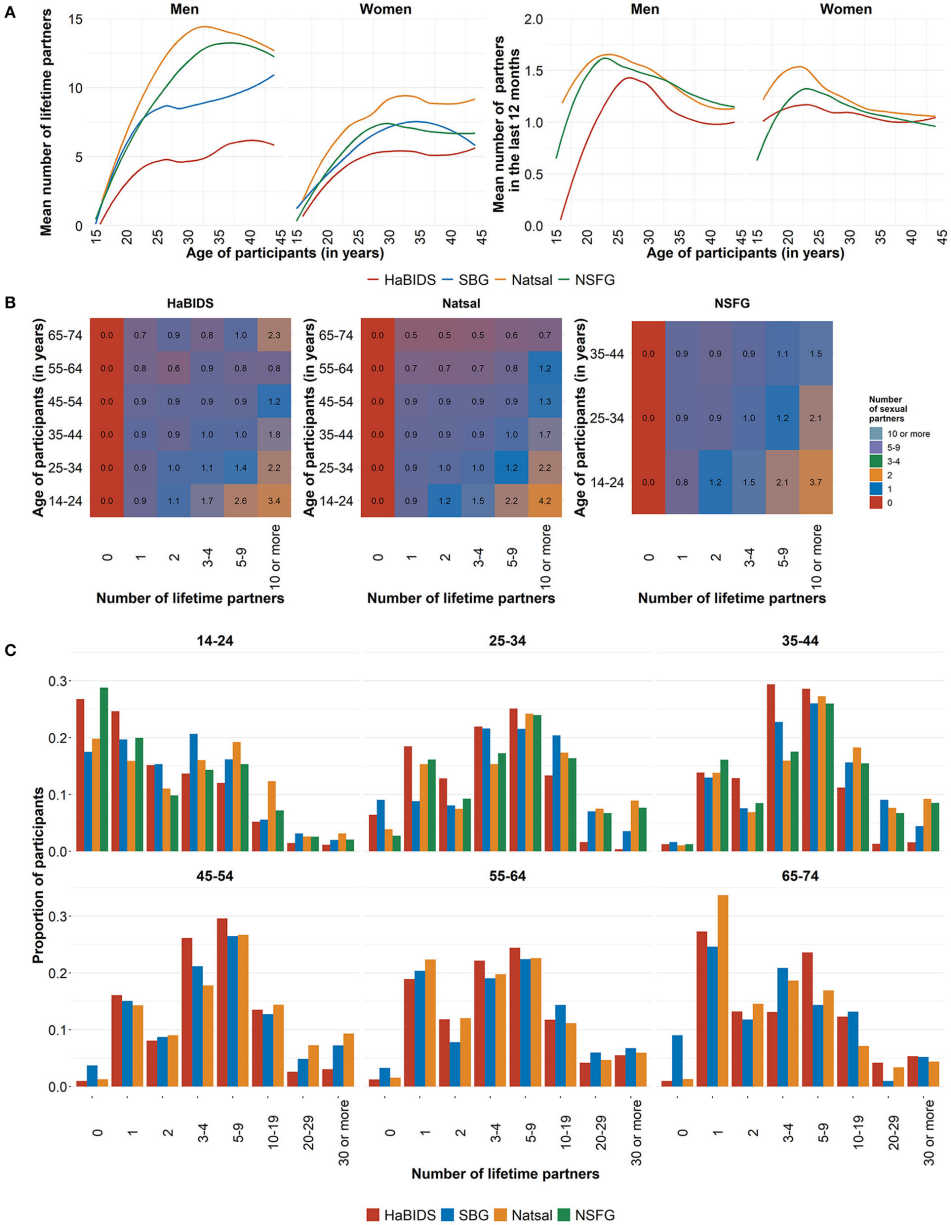
Comparison between studies on **(A)** the mean number of reported opposite-sex sexual partners by age in a lifetime and in the last 12 months; **(B)** mean number of opposite-sex sexual partners in the last 12 months by age and lifetime partners; **(C)** proportion of lifetime partners across age groups.

The difference in lifetime partners can be linked to differences in the number of partners in younger age groups (14–24 years), while in the older age groups, the age-related increase in the number of partners was similar. Consequently, the acquisition of new partners (partners in the last 12 months) differed more in younger age groups across countries (Figure 4A, right panel). These patterns were generally similar in men and women, although absolute numbers of partners were consistently higher in men. The higher acquisition rate of new partners was not evenly distributed across all participants but caused by a higher proportion of persons in the groups with the highest partner changes (10–19, 20–29, 30 or more) in Natsal and, to a lesser extent, in NSFG (Figure 4C). Those who reported more lifetime sexual partners also had more sexual partners in the last 12 months, supporting individual stability of behavior as used in the concept of stable sexual activity groups (Figure 4B).

Apart from the younger age groups (below 25 years for women and 30 years for men), the proportions of both men and women with a given number of lifetime partners were stable across age. The number of reported lifetime partners was highest in Natsal followed by NSFG and both German studies (Figure 4A, left panel, Table 3).

**Table 3:** Comparison of the number of sexual partners between studies adjusted for age and sex.

**(A). Without age restrictions in HaBIDS, SBG, and Natsal**.			
**IRR (95% CI; weighted negative binomial regression)**	**SBG**	**Natsal**	
Lifetime opposite-sex partners	1.22 (1.12, 1.34)	1.67 (1.55, 1.79)	
Opposite-sex partners in the last 12 months	-	1.08 (1.01, 1.15)	
New opposite-sex partners in the last 12 months	-	1.15 (0.96, 1.38)	
Lifetime same-sex partners	1.02 (0.65, 1.58)	1.00 (0.68, 1.42)	
Lifetime same-sex partners (at least one same-sex partner)	1.14 (0.82, 1.58)	1.85 (1.40, 2.42)	
Same-sex partners in the last 12 months	-	1.01 (0.70, 1.45)	
New same-sex partners in the last 12 months		1.37 (0.98, 1.93)	
Both-sex sexual partners in the entire life	2.14 (1.67, 2.73)	2.72 (2.23, 3.29)	
Both-sex sexual partners in the last 12 months	-	1.30 (1.05, 1.62)	
New sexual partners (both-sex) in the last 12 months	-	1.87 (1.04, 3.18)	
**(B). Restricting HaBIDS, SBG, and Natsal participants' age to 45 years (NSFG maximum age)**.			
**IRR (95% CI; weighted negative binomial regression)**	**SBG**	**NSFG**	**Natsal**
Lifetime opposite-sex partners (all age group)	1.43 (1.27, 1.71)	1.64 (1.49, 1.80)	2.05 (1.86, 2.25)
Lifetime opposite-sex partners (14–24 years)	1.52 (1.18, 1.95)	1.47 (1.20, 1.79)	1.99 (1.62, 2.43)
Lifetime opposite-sex partners (25–34 years)	1.63 (1.34, 1.98)	1.83 (1.56, 2.14)	2.23 (1.89, 2.61)
Lifetime opposite-sex partners (35–44 years)	1.53 (1.28, 1.82)	1.65 (1.43, 1.90)	1.98 (1.71, 2.29)
Opposite-sex partners in the last 12 months	-	1.04 (0.96, 1.13)	1.17 (1.08, 1.28)
Lifetime same-sex partners	0.64 (0.36, 1.10)	0.28 (0.18, 0.43)	0.76 (0.47, 1.15)
Lifetime same-sex partners (at least one same-sex partner)	0.72 (0.49, 1.04)	0.48 (0.35, 0.65)	1.43 (1.03, 1.95)
Same-sex partners in the last 12 months	-	0.55 (0.36, 0.84)	1.11 (0.71, 1.71)
Both-sex sexual partners in the entire life	1.56 (1.17, 2.07)	1.70 (1.35, 2.11)	2.73 (2.16, 3.43)
Both-sex sexual partners in the last 12 months	-	0.84 (0.65, 1.10)	1.47 (1.12, 1.93)

*HaBIDS serves as the reference study.*

*Opposite/same/both-sex partners in the last 12 months are based on participants who had at least one lifetime opposite/same/both-sex sexual partner. New opposite/same/both-sex sexual partners in the last 12 months are based on participants who had at least one opposite/same/both-sex sexual partner in the last 12 months.*

*CI, confidence interval; IRR, incidence rate ratio; OR, odds ratio*.

#### Opposite-Sex Partners in the Last 12 Months

Among HaBIDS participants who had at least one lifetime opposite-sex sexual partner, 14% reported to have had no opposite-sex sexual partner in the last 12 months, while 79% reported one and 7% reported two or more opposite-sex sexual partners in the last 12 months. Among the 15–19 years old HaBIDS participants, among those with at least one lifetime opposite-sex partner, all women had at least one opposite-sex partner in the last 12 months, whereas 25% of men had no opposite-sex sexual partner in the last 12 months (Figure 3B). While there was no evidence for overall differences between HaBIDS and Natsal participants (IRR 1.08, 95%CI 1.01–1.15) (Table 3A; Figure 4B; Supplementary Table 3), Natsal participants in the age group 14–24 years showed a higher number of opposite-sex sexual partner in the last 12 months (IRR 1.26, 95%CI 1.07–1.50) consistent with the analysis on lifetime partners.

#### New Opposite-Sex Partners in the Last 12 Months

Of those participants in HaBIDS with at least one opposite-sex sexual partner in the last 12 months, only 19% reported one or more new opposite-sex sexual partners in the last 12 months. The highest proportion of participants who reported a new opposite-sex sexual partner occurred in the age group 15–19 years (88%) (Figure 3C). Natsal reported a slightly higher number of new opposite-sex sexual partners (IRR 1.15, 95%CI 0.96–1.38) among those who had at least one lifetime partner compared to HaBIDS (Table 3A; Figure 4B; Supplementary Table 4).

### Same-Sex Partners During Lifetime and in the Last 12 Months

**Figure 5:**
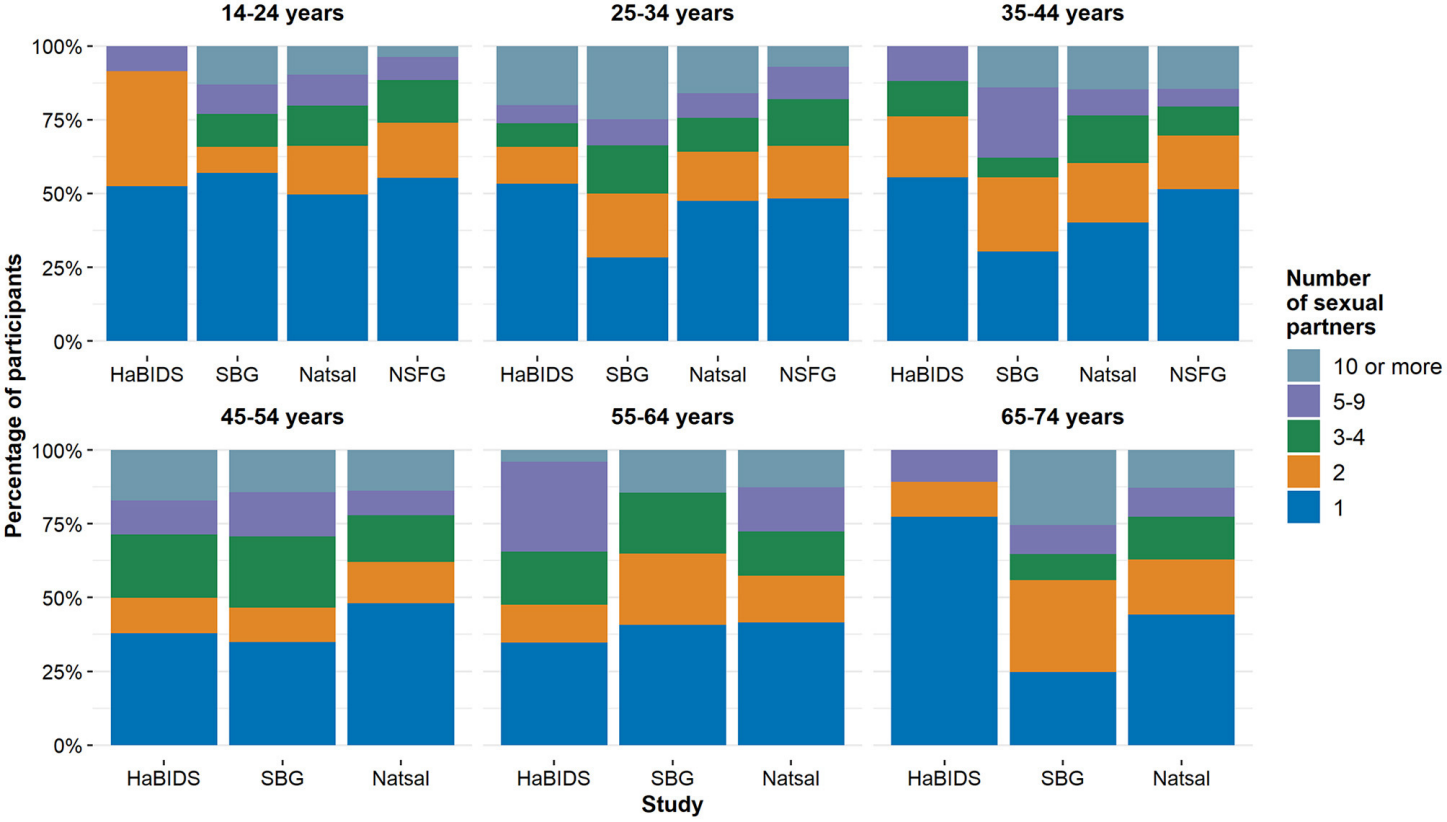
Comparison between studies on those who reported to have had at least one same-sex lifetime sexual partner categorized by age.

Within HaBIDS, 121 participants (11%; 38 men, 83 women) reported at least one same-sex sexual partner in their lifetime (in addition to potential opposite-sex sexual partners). Among participants who reported at least one same-sex sexual partnership, HaBIDS participants had a median of two lifetime same-sex partners (IQR 1–2). The number of partners was similar in SBG (IRR 1.14, 95%CI 0.82–1.58) and higher in Natsal (IRR 1.85, 95%CI 1.40–2.42; Figure 5; Table 3A).

The same was true for Natsal (IRR 1.43, 95%CI 1.03–1.95) when the participant's age was restricted to NSFG maximum age range. However, SBG (IRR 0.72, 95%CI 0.49–1.04) and NSFG (IRR 0.48, 95%CI 0.35–0.65; Table 3B) reported lower number of same-sex partners.

Fifty percent of HaBIDS participants with at least one lifetime same-sex sexual partner reported one or more same-sex sexual partner in the last 12 months (Supplementary Table 6). NSFG participants reported lower numbers of same-sex sexual partners in the last 12 months compared to HaBIDS (IRR 0.55, 95%CI 0.36–0.84); this was not the case for Natsal (IRR 1.11, 95%CI 0.71–1.71; Table 3B). In comparison to HaBIDS, Natsal reported slightly higher number of new same-sex partners among those participants with a same-sex sexual partner in the past 12 months (IRR 1.37, 95%CI 0.98–1.93; Table 3; Supplementary Table 7).

### Participants With Both-Sex Sexual Experience During Lifetime and in the Last 12 Months

One hundred and thirteen (9%) of the HaBIDS participants reported to have had at least one opposite- and one same-sex sexual partner at some point during their lives. This percentage was similar in NSFG (10%) but considerably lower in Natsal (5%) and SBG (6%). Among those participants with both-sex sexual experience, HaBIDS participants reported to have had a median of seven lifetime partners (IQR 6–8; Supplementary Table 8). The number of lifetime partners was higher in the other three studies (NSFG IRR 1.70, 95%CI 1.35–2.11; SBG IRR 1.56, 95%CI 1.17–2.07; Natsal IRR 2.73, 95%CI 2.16–3.43; Table 3B).

## Discussion

In this manuscript we provide for the first time systematically collected data on sexual contact patterns in the German population from two independent studies; these data can be used for the parametrization of mathematical models in the field of sexually transmissible infections which focus specifically on the German situation. This is specifically important when evaluating the potential population-wide effects of prevention measures targeting the transmission of STIs in the country e.g., during the introduction of the vaccination program against Human Papilloma Viruses 10 years ago. The identified lack of sexual contact data during the modeling studies supporting the evaluation of the potential effects of the introduction of the program in Germany were the motivation for setting up the HaBIDS sexual contact survey and for this comparison study. The parameterisation of the respective model at that time was based on Natsal data (21). Knowledge about country-specific sexual contact patterns would have helped to understand if the effects of the vaccination program predicted in this modeling study would have changed, e.g., when the number of sexual contacts would have been considerably lower in Germany than in the UK. Our analysis found that sexual contact patterns in Germany were in general similar to those reported from the UK and US. However, there were key differences with respect to the proportion of participants with a high number of partners in younger age groups or the proportion of individuals with both-sex sexual experience. The results of both German studies agreed in the majority of analyses.

The median age of the participants' first sexual contact in HaBIDS was 17 years in women and 18 years in men which is consistent with the median age of sexual debut in Natsal and NSFG (7, 8). Across all age groups, over three-fourths of participants in HaBIDS and Natsal maintained a stable opposite-sex sexual relationship in the last 12 months. The only exceptions were those below 25 years, where almost half of the study population in both studies reported one or more new opposite-sex sexual partners in the last 12 months. This is not unexpected, as research has previously demonstrated that adolescents are more likely than adults to report multiple sex partners in the recent past (22, 23).

German studies showed a lower number of lifetime sexual partners than Natsal and NSFG. This pattern was driven by a higher proportion of individuals with a high number of partners in the younger age groups in the UK and US, while both age of sexual debut and sexual contact patterns in older ages did not differ between countries. The observed differences have potentially huge implications for the way infectious diseases spread in the respective societies, since high activity groups are at the core of transmission. This means that the observed differences might be more important for modeling studies focusing on STIs where these early contacts are crucial for overall transmission dynamics and burden of disease as it is the case for the Human Papilloma Virus (HPV). In contrast, the observed differences might affect the modeling of those STIs to a lesser extent which are transmitted throughout life and for which transmissions in all age groups are equally important for overall infection dynamics as it is the case for chlamydia or gonorrhea.

Less than 10% of all participants reported to have ever had a same-sex sexual relationship in all studies across all age groups; among those with experience with sexual partners from both sexes, the number of lifetime sexual partners was considerably lower in HaBIDS than in the other studies. This is especially important because this subpopulation serves as a bridging population in diseases where transmission dynamics are different among opposite-sex and same-sex partnerships.

Even though the four surveys were conducted independently with different setups, they sought the same information. Nevertheless, the questions used in the four surveys differed slightly from each other, opening the chance for misclassification. This is often the case when comparing results from various surveys and needs to be kept in mind in future studies comparing survey results as well.

Some of the key dissimilarities observed between the studies designs are differences in sampling techniques, survey aims, or the longevity of projects associated with surveys. More sexual partners were reported in the UK, which could be attributed to Natsal's long-term research on sexual contacts, so that study participants were aware and familiar with Natsal and less likely to report lower numbers of partners because of societal norms. On the contrary, HaBIDS was not purely designed to focus on sexual behavior but evaluated in more general terms knowledge, attitudes, and practice related to infectious diseases so that participants might have felt less comfortable and might have underreported the true number of sexual partners.

The data were cross-sectional so that comparisons across age are also comparisons of different age cohorts. Nevertheless, the observed patterns across age appeared consistent with stable partner change behavior in the last few decades.

The long-term panel nature of HaBIDS resulted in a much lower response proportion (9%) compared with SBG (52%), Natsal (58%), and NSFG (77%); while willingness to participate in studies generally decreases, the lower response proportion could be a potential source of bias e.g., because individuals with University degrees were more likely to participate in HaBIDS, and tend to differ in their sexual contact patterns from individuals without University degrees. We tried to address this by weighing study populations in a way that they became representative of the source population. HaBIDS and SBG were weighted by age and sex to get a representative sample of the country's population. Natsal chose a similar approach and compensated for oversampling the younger participants aged 16–34 years by using probability weights from the 2011 UK census data based on age, sex and government office region (7, 16). More sophisticated sampling weights were provided for NSFG data to account for the complex sample design and estimation features of the survey (8). Harling et al. investigated the effect of weighting for sampling and non-response on the estimates of STI prevalence in Natsal-3 (24). They were able to show that accounting for sampling and non-response does indeed affects national STI prevalence estimates heavily. Unweighted Natsal estimates were strongly biased toward an overestimation of estimates whereas estimates based on weighted data as it was done in our comparison study offer a rather unbiased estimate of true behavior in the population. Additionally, the sample sizes in the youngest and the oldest, i.e., participants aged <20 years and >65 years, are rather small especially in the HaBIDS study. So even after applying weights, this should be kept in mind when interpreting the results of the youngest and oldest study groups.

In all the four studies, participants filled in questionnaires by themselves without interviewers. While this provided more privacy, it is still possible that respondents deliberately chose not to divulge correct information. Moreover, respondents might not have recalled information correctly, particularly if the numbers were high. In case of difficulties to recall, partnerships in the past 12 months may be the most reliable measure (25). Given that these points affected all studies equally, it is unlikely that they were responsible for observed differences between studies.

In both German studies, there was a lower proportion of participants with a high number of partners in younger age groups when compared to Natsal and NSFG. This resulted in a lower number of lifetime partners also at older ages, even if the partner acquisition above 24 years was similar in all studied populations. Dependent on the characteristics of the studied sexually transmitted infections, these patterns can lead to substantially different prevalence and incidences of sexually transmitted infections across ages and have a strong impact on modeling studies of potential interventions (e.g., vaccination or screening effects).

## Data Availability Statement

The raw data supporting the conclusions of this article will be made available by the authors, without undue reservation.

## Ethics Statement

HaBIDS was approved by the Ethics Committee of Hannover Medical School (No. 2021-2013) and by the Federal Commissioner for Data Protection and Freedom of Information in Germany. All participants provided written informed consent prior to study entry. Informed consent was obtained from a parent or guardian on behalf of any participants aged 18 or below. Written informed consent to participate in this study was provided by the participants' legal guardian/next of kin.

## Author Contributions

NR, AK, and RM designed the HaBIDS study. CK designed and performed the SBG study. DT, VJ, SK, NR, and JH conducted the statistical analyses. DT, VJ, AK, and RM wrote the manuscript. All authors interpreted the study findings, contributed to the manuscript, and approved the final version of the manuscript.

## Conflict of Interest

The authors declare that the research was conducted in the absence of any commercial or financial relationships that could be construed as a potential conflict of interest.

## Publisher's Note

All claims expressed in this article are solely those of the authors and do not necessarily represent those of their affiliated organizations, or those of the publisher, the editors and the reviewers. Any product that may be evaluated in this article, or claim that may be made by its manufacturer, is not guaranteed or endorsed by the publisher.

## References

[1] WHO Department of Reproductive Health and Research. Report on Global Sexually Transmitted Infection Surveillance, 2018. World Health Organization (2018).

[2] Hughes G Field N. The epidemiology of sexually transmitted infections in the UK: impact of behavior, services and interventions. Future Microbiol. (2015) 10:35–51. 10.2217/fmb.14.110 25598336

[3] Aral SO, Over, M, Manhart, L, Holmes, KK,. Disease Control Priorities in Developing Countries, Chapter 17: Sexually Transmitted Infections. 2nd ed. (2006). p. 311–30. Available online at: http://www.who.int/management/referralhospitals.pdf. 10.1596/978-0-8213-6179-5/Chpt-17 (accessed September 21, 2021).

[4] Lurie MN Rosenthal S. Concurrent partnerships as a driver of the HIV epidemic in sub-saharan Africa? The evidence is limited. AIDS Behav. (2010) 14:17–24. 10.1007/s10461-009-9583-5 19488848 PMC3819098

[5] Díaz A Díez M. Infecciones de transmisión sexual: epidemiología y control. Revista Española de Sanidad Penitenciaria. (2011) 13:58–66. 10.4321/S1575-06202011000200005 21750856

[6] Mabey D. Epidemiology of sexually transmitted infections: worldwide. Medicine. (2014) 42:287–90. 10.1016/j.mpmed.2014.03.004

[7] Mercer CH Tanton C Prah P Erens B Sonnenberg P Clifton S. Changes in sexual attitudes and lifestyles in Britain through the life course and over time: findings from the National Surveys of Sexual Attitudes and Lifestyles (Natsal). Lancet. (2013) 382:1781–94. doi,: 10.1016/S.0140-6736(13)62035-8 24286784 10.1016/S0140-6736(13)62035-8 PMC3899021

[8] Lepkowski JM Mosher WD Davis KE Groves RM Van Hoewyk J. The 2006-2010 National Survey of Family Growth: Sample Design and Analysis of a Continuous Survey. Vital Health Stat. (2010) 1–36. 20928970

[9] Chandra A Mosher WD Copen C Sionean C. Sexual behavior, sexual attraction, and sexual identity in the united states: data from the 2006-2008 national survey of family growth. Sexual Statistics. (2013) 19:1–74. 10.1007/978-94-007-5512-3_4 21560887

[10] Rübsamen N Akmatov MK Castell S Karch A Mikolajczyk RT. Comparison of response patterns in different survey designs: a longitudinal panel with mixed-mode and online-only design. Emerg Themes Epidemiol. (2017) 14:1–11. 10.1186/s12982-017-0058-2 28344629 PMC5361716

[11] Rübsamen N Castell S Horn J Karch A Ott JJ Raupach-Rosin H. Ebola risk perception in Germany, 2014. Emerg Infect Dis. (2015) 21:150013. 10.3201/eid2106.150013 25989020 PMC4451905

[12] Rübsamen N Akmatov MK Castell S Karch A Mikolajczyk RT. Factors associated with attrition in a longitudinal online study: results from the HaBIDS panel. BMC Med Res Methodol. (2017) 17:1–11. 10.1186/s12874-017-0408-3 28859617 PMC5580321

[13] Haversath J Gärttner KM Kliem S Vasterling I Strauss B Kröger C. Sexual behavior in Germany - results of a representative survey. Dtsch Arztebl Int. (2017) 114:545–50. 10.3238/arztebl.2017.0545 28855044 PMC5596148

[14] Lepkowski JM Mosher WD Groves RM West BT Wagner J Gu H. Responsive Design, Weighting, and Variance Estimation in the 2006-2010 National Survey of Family Growth. Vital Health Stat. (2013) 1–52. 25093250

[15] Zensus. Bevölkerungs- und Wohnungszählung. (2011). Available online at: https://www.zensus2011.de/DE/Zensus2011/zensus2011_node.html (accessed September 21, 2021).

[16] Erens B, Phelps, A, Clifton, S, Hussey, D, Mercer, CH, Tanton, C, . National Survey of Sexual Attitudes Lifestyles 3 Technical Report - Volume 1: Methodology. Natsal. (2013). p. 56. Available online at: http://www.natsal.ac.uk/media/2090/natsal-3-technical-report.pdf (accessed September 21, 2021).

[17] R Development Team,. A Language Environment for Statistical Computing. Vienna: R Foundation for Statistical Computing (2019). Available online at: https://www.r-project.org/ (accessed September 21, 2021).

[18] Wickham H,. ggplot2: Elegant Graphics for Data Analysis. New York, NY: Springer-Verlag. (2016). Available online at: https://ggplot2.tidyverse.org (accessed September 21, 2021).

[19] Lumley T. Survey: *Analysis of complex survey samples*. R Package Version 4.0 (2020).

[20] Venables WN Ripley BD. Modern Applied Statistics With S. New York, NY: Springer. (2002). 10.1007/978-0-387-21706-2

[21] Horn J Damm O Kretzschmar MEE Deleré Y Wichmann O Kaufmann AM. Estimating the long-term effects of HPV vaccination in Germany. Vaccine. (2013) 31:2372–80. 10.1016/j.vaccine.2013.03.006 23518405

[22] Durbin M DiClemente RJ Siegel D Krasnovsky F Lazarus N Camacho T. Factors associated with multiple sex partners among junior high school students. J Adolesc Health. (1993) 14:202–7. 10.1016/1054-139X(93)90006-B 8323931

[23] Santelli JS Brener ND Lowry R Bhatt A Zabin LS. Multiple sexual partners among U.S. Adolescents and young adults. Family Plan Perspectiv. (1998) 30:271–5. 10.2307/2991502 9859017

[24] Harling G Copas A Clifton S Johnson AM Field N Sonnenberg P. Effect of weighting for sampling and non-response on estimates of STI prevalence in the third British National Survey of Sexual Attitudes and Lifestyles (Natsal-3). Sex Transm Infect. (2020) 96:481–4. 10.1136/sextrans-2019-054342 32220980 PMC7591710

[25] Todd J Cremin I McGrath N Bwanika JB Wringe A Marston M. Reported number of sexual partners: comparison of data from four African longitudinal studies. Sexually Transmitted Infect. (2009) 85(suppl.1):33985. 10.1136/sti.2008.033985 PMC2654146 19307344

