# Supplementary material for: Sexual Contact Patterns in High-Income Countries—A Comparative Analysis Using Data From Germany, the United Kingdom, and the United States
Source: Front Epidemiol. 2022 May 4;2:858789. doi: 10.3389/fepid.2022.858789 (PMC10911028; doi:10.3389/fepid.2022.858789)
Supplement: Supplementary file 1 [file Data_Sheet_1.docx]

**Table 1: Investigate the impact of different modes of questionnaires (paper versus online) on the reported number of sexual partners in HaBIDS using weighted negative binomial regression taking age, sex, and education into account.**

Number of sexual partners in the last 12 months is based on participants who had at least one lifetime opposite/same-sex sexual partner. Number of new sexual partners in the last 12 months is based on participants who had at least one opposite/same-sex sexual partner in the last 12 months. The online version of the questionnaire is the reference category.

CI, confidence interval; IRR, Incidence rate ratio

|  | **IRR** | **95%CI** |
| --- | --- | --- |
| Opposite-sex partners in the entire life | 0.94 | 0.82, 1.07 |
| Opposite-sex partners in the last 12 months | 1.10 | 0.97, 1.25 |
| New opposite-sex partners in the last 12 months | 1.68 | 1.24, 2.29 |
| Same-sex partners in the entire life | 0.74 | 0.40, 1.41 |
| Same-sex partners in the last 12 months | 0.56 | 0.31, 1.01 |

**Table 2: Frequency of reported number of lifetime opposite-sex sexual partners by age group and study**

IQR, interquartile range.

**Table 2a: Participants aged 14 to 24 years**

|  | **HaBIDS** | | **SBG** | | **Natsal** | | **NSFG** | |
| --- | --- | --- | --- | --- | --- | --- | --- | --- |
| **Number of sexual partners** | **Men (n=39)** | **Women (n=58)** | **Men (n=128)** | **Women (n=135)** | **Men (n=1701)** | **Women (n=2105)** | **Men (n=3971)** | **Women (n=4270)** |
| 0 | 13 (33.3%) | 7 (12.1%) | 37 (28.9%) | 25 (18.5%) | 364 (21.4%) | 396 (18.8%) | 1143 (28.8%) | 1142 (26.7%) |
| 1 | 8 (20.5%) | 16 (27.6%) | 23 (18.0%) | 26 (19.3%) | 273 (16.0%) | 336 (16.0%) | 696 (17.5%) | 916 (21.5%) |
| 2 | 6 (15.4%) | 10 (17.2%) | 18 (14.1%) | 22 (16.3%) | 185 (10.9%) | 219 (10.4%) | 363 (9.1%) | 469 (11.0%) |
| 3-4 | 5 (12.8%) | 10 (17.2%) | 18 (14.1%) | 31 (23.0%) | 248 (14.6%) | 348 (16.5%) | 564 (14.2%) | 650 (15.2%) |
| 5-9 | 3 (7.7%) | 11 (19.0%) | 20 (15.6%) | 20 (14.8%) | 300 (17.6%) | 447 (21.2%) | 627 (15.8%) | 690 (16.2%) |
| 10 or more | 4 (10.3%) | 4 (6.9%) | 12 (9.4%) | 11 (8.1%) | 331 (19.5%) | 359 (17.1%) | 578 (14.6%) | 403 (9.4%) |
| Mean | 3 | 4 | 5 | 4 | 7 | 5 | 5 | 3 |
| Median [IQR] | 1 [0-3] | 2 [1-4] | 2 [1-5] | 2 [1-4] | 3 [1-7] | 3 [1-7] | 2 [0-5] | 1 [0-4] |

**Table 2b: Participants aged 25 to 34 years**

|  | **HaBIDS** | | **SBG** | | **Natsal** | | **NSFG** | |
| --- | --- | --- | --- | --- | --- | --- | --- | --- |
| **Number of sexual partners** | **Men (n=56)** | **Women (n=126)** | **Men (n=160)** | **Women (n=183)** | **Men (n=1477)** | **Women (n=2412)** | **Men (n=3266)** | **Women (n=4330)** |
| 0 | 7 (12.5%) | 2 (1.6%) | 11 (6.9%) | 19 (10.4%) | 70 (4.7%) | 53 (2.2%) | 133 (4.1%) | 115 (2.7%) |
| 1 | 8 (14.3%) | 29 (23.0%) | 13 (8.1%) | 18 (9.8%) | 160 (10.8%) | 384 (15.9%) | 384 (11.8%) | 797 (18.4%) |
| 2 | 6 (10.7%) | 17 (13.5%) | 8 (5.0%) | 20 (10.9%) | 92 (6.2%) | 196 (8.1%) | 228 (7.0%) | 457 (10.6%) |
| 3-4 | 14 (25.0%) | 25 (19.8%) | 30 (18.8%) | 46 (25.1%) | 209 (14.2%) | 398 (16.5%) | 473 (14.5%) | 826 (19.1%) |
| 5-9 | 13 (23.2%) | 31 (24.6%) | 39 (24.4%) | 34 (18.6%) | 354 (24.0%) | 628 (26.0%) | 761 (23.3%) | 1115 (25.8%) |
| 10 or more | 8 (14.3%) | 22 (17.5%) | 59 (36.9%) | 46 (25.1%) | 592 (40.1%) | 753 (31.2%) | 1287 (39.4%) | 1020 (23.6%) |
| Mean | 5 | 5 | 9 | 7 | 14 | 9 | 11 | 7 |
| Median [IQR] | 3 [1-7] | 4 [1-6] | 6 [3-11] | 4 [2-10] | 6 [3-15] | 5 [2-10] | 6 [3-15] | 4 [2-9] |

**Table 2c: Participants aged 35 to 44 years**

|  | **HaBIDS** | | **SBG** | | **Natsal** | | **NSFG** | |
| --- | --- | --- | --- | --- | --- | --- | --- | --- |
| **Number of sexual partners** | **Men (n=76)** | **Women (n=131)** | **Men (n=146)** | **Women (n=184)** | **Men (n=787)** | **Women (n=1168)** | **Men (n=2822)** | **Women (n=3390)** |
| 0 | 2 (2.6%) | 0 (0%) | 3 (2.1%) | 3 (1.6%) | 12 (1.5%) | 5 (0.4%) | 75 (2.7%) | 42 (1.2%) |
| 1 | 10 (13.2%) | 19 (14.5%) | 17 (11.6%) | 24 (13.0%) | 82 (10.4%) | 160 (13.7%) | 263 (9.3%) | 594 (17.5%) |
| 2 | 9 (11.8%) | 18 (13.7%) | 6 (4.1%) | 19 (10.3%) | 38 (4.8%) | 92 (7.9%) | 166 (5.9%) | 339 (10.0%) |
| 3-4 | 23 (30.3%) | 38 (29.0%) | 19 (13.0%) | 58 (31.5%) | 95 (12.1%) | 225 (19.3%) | 374 (13.3%) | 687 (20.3%) |
| 5-9 | 21 (27.6%) | 38 (29.0%) | 42 (28.8%) | 45 (24.5%) | 204 (25.9%) | 334 (28.6%) | 662 (23.5%) | 975 (28.8%) |
| 10 or more | 11 (14.5%) | 18 (13.7%) | 59 (40.4%) | 35 (19.0%) | 356 (45.2%) | 352 (30.1%) | 1282 (45.4%) | 753 (22.2%) |
| Mean | 6 | 5 | 11 | 7 | 14 | 9 | 13 | 7 |
| Median [IQR] | 4 [2-6] | 4 [2-6] | 8 [3-15] | 4 [2-7] | 8 [4-18] | 5 [3-10] | 7 [3-16] | 4 [2-8] |

**Table 2d: Participants aged 45 to 54 years**

|  | **HaBIDS** | | **SBG** | | **Natsal** | |
| --- | --- | --- | --- | --- | --- | --- |
| **Number of sexual partners** | **Men (n=91)** | **Women (n=200)** | **Men (n=200)** | **Women (n=227)** | **Men (n=757)** | **Women (n=1066)** |
| 0 | 2 (2.2%) | 0 (0%) | 5 (2.5%) | 10 (4.4%) | 19 (2.5%) | 12 (1.1%) |
| 1 | 13 (14.3%) | 37 (18.5%) | 22 (11.0%) | 38 (16.7%) | 60 (7.9%) | 170 (15.9%) |
| 2 | 7 (7.7%) | 15 (7.5%) | 13 (6.5%) | 24 (10.6%) | 50 (6.6%) | 104 (9.8%) |
| 3-4 | 23 (25.3%) | 53 (26.5%) | 32 (16.0%) | 57 (25.1%) | 95 (12.5%) | 236 (22.1%) |
| 5-9 | 27 (29.7%) | 59 (29.5%) | 50 (25.0%) | 63 (27.8%) | 192 (25.4%) | 311 (29.2%) |
| 10 or more | 19 (20.9%) | 36 (18.0%) | 78 (39.0%) | 35 (15.4%) | 341 (45.0%) | 233 (21.9%) |
| Mean | 6 | 7 | 15 | 6 | 18 | 7 |
| Median [IQR] | 4 [2-7] | 4 [2-8] | 5 [3-12] | 4 [2-7] | 8 [4-20] | 4 [2-7] |

**Table 2e: Participants aged 55 to 64 years**

|  | **HaBIDS** | | **SBG** | | **Natsal** | |
| --- | --- | --- | --- | --- | --- | --- |
| **Number of sexual partners** | **Men (n=113)** | **Women (n=134)** | **Men (n=176)** | **Women (n=224)** | **Men (n=709)** | **Women (n=984)** |
| 0 | 2 (1.8%) | 1 (0.7%) | 5 (2.8%) | 9 (4.0%) | 25 (3.5%) | 16 (1.6%) |
| 1 | 19 (16.8%) | 28 (20.9%) | 27 (15.3%) | 53 (23.7%) | 95 (13.4%) | 261 (26.5%) |
| 2 | 9 (8.0%) | 21 (15.7%) | 4 (2.3%) | 28 (12.5%) | 61 (8.6%) | 136 (13.8%) |
| 3-4 | 29 (25.7%) | 25 (18.7%) | 26 (14.8%) | 48 (21.4%) | 115 (16.2%) | 220 (22.4%) |
| 5-9 | 24 (21.2%) | 37 (27.6%) | 44 (25.0%) | 47 (21.0%) | 174 (24.5%) | 218 (22.2%) |
| 10 or more | 30 (26.5%) | 22 (16.4%) | 70 (39.8%) | 39 (17.4%) | 239 (33.7%) | 133 (13.5%) |
| Mean | 12 | 6 | 11 | 6 | 20 | 6 |
| Median [IQR] | 4 [2-10] | 4 [2-7] | 6 [3-12] | 3 [1-7] | 5 [2-11] | 3 [1-5] |

**Table 2f: Participants aged 65 to 74 years**

|  | **HaBIDS** | | **SBG** | | **Natsal** | |
| --- | --- | --- | --- | --- | --- | --- |
| **Number of sexual partners** | **Men (n=44)** | **Women (n=54)** | **Men (n=145)** | **Women (n=135)** | **Men (n=595)** | **Women (n=793)** |
| 0 | 0 (0%) | 1 (1.9%) | 11 (7.6%) | 14 (10.4%) | 13 (2.2%) | 10 (1.3%) |
| 1 | 9 (20.5%) | 18 (33.3%) | 20 (13.8%) | 46 (34.1%) | 130 (21.8%) | 324 (40.9%) |
| 2 | 5 (11.4%) | 8 (14.8%) | 16 (11.0%) | 16 (11.9%) | 65 (10.9%) | 140 (17.7%) |
| 3-4 | 4 (9.1%) | 9 (16.7%) | 31 (21.4%) | 29 (21.5%) | 108 (18.2%) | 156 (19.7%) |
| 5-9 | 12 (27.3%) | 11 (20.4%) | 25 (17.2%) | 15 (11.1%) | 135 (22.7%) | 96 (12.1%) |
| 10 or more | 14 (31.8%) | 7 (13.0%) | 42 (29.0%) | 15 (11.1%) | 144 (24.2%) | 67 (8.4%) |
| Mean | 22 | 4 | 11 | 4 | 12 | 6 |
| Median [IQR] | 5 [2-12] | 2 [1-5] | 4 [2-10] | 2 [1-4] | 4 [2-8] | 2 [1-4] |

**Table 3: Frequency of reported number of opposite-sex sexual partners in the last 12 months by age group and study based on participants who had at least one lifetime opposite-sex sexual partner****.**

IQR, interquartile range.

**Table 3a: Participants aged 14 to 24 years**

|  | **HaBIDS** | | **Natsal** | | **NSFG** | |
| --- | --- | --- | --- | --- | --- | --- |
| **Number of sexual partners** | **Men (n=25)** | **Women (n=50)** | **Men (n=1328)** | **Women (n=1700)** | **Men (n=2812)** | **Women (n=3122)** |
| 0 | 3 (12.0%) | 1 (2.0%) | 76 (5.7%) | 59 (3.5%) | 294 (10.5%) | 236 (7.6%) |
| 1 | 15 (60.0%) | 35 (70.0%) | 687 (51.7%) | 1050 (61.8%) | 1406 (50.0%) | 1903 (61.0%) |
| 2 or more | 7 (28.0%) | 14 (28.0%) | 565 (42.5%) | 591 (34.8%) | 1112 (39.5%) | 983 (31.5%) |
| Mean | 1 | 2 | 2 | 2 | 2 | 2 |
| Median [IQR] | 1 [1-2] | 1 [1-2] | 1 [1-2] | 1 [1-2] | 1 [1-2] | 1 [1-2] |

**Table 3b: Participants aged 25 to 34 years**

|  | **HaBIDS** | | **Natsal** | | **NSFG** | |
| --- | --- | --- | --- | --- | --- | --- |
| **Number of sexual partners** | **Men (n=49)** | **Women (n=122)** | **Men (n=1404)** | **Women (n=2353)** | **Men (n=3123)** | **Women (n=4214)** |
| 0 | 3 (6.1%) | 4 (3.3%) | 80 (5.7%) | 155 (6.6%) | 225 (7.2%) | 297 (7.0%) |
| 1 | 35 (71.4%) | 109 (89.3%) | 1005 (71.6%) | 1849 (78.6%) | 2142 (68.6%) | 3229 (76.6%) |
| 2 or more | 11 (22.4%) | 9 (7.4%) | 319 (22.7%) | 349 (14.8%) | 756 (24.2%) | 688 (16.3%) |
| Mean | 1 | 1 | 2 | 1 | 2 | 1 |
| Median [IQR] | 1 [1-1] | 1 [1-1] | 1 [1-1] | 1 [1-1] | 1 [1-1] | 1 [1-1] |

**Table 3c: Participants aged 35 to 44 years**

|  | **HaBIDS** | | **Natsal** | | **NSFG** | |
| --- | --- | --- | --- | --- | --- | --- |
| **Number of sexual partners** | **Men** (n=74) | **Women** (n=126) | **Men** (n=772) | **Women** (n=1159) | **Men** (n=2745) | **Women** (n=3345) |
| 0 | 6 (8.1%) | 10 (7.9%) | 66 (8.5%) | 128 (11.0%) | 324 (11.8%) | 443 (13.2%) |
| 1 | 63 (85.1%) | 107 (84.9%) | 596 (77.2%) | 917 (79.1%) | 1931 (70.3%) | 2593 (77.5%) |
| 2 or more | 5 (6.8%) | 9 (7.1%) | 110 (14.2%) | 114 (9.8%) | 490 (17.9%) | 309 (9.2%) |
| Mean | 1 | 1 | 1 | 1 | 1 | 1 |
| Median [IQR] | 1 [1-1] | 1 [1-1] | 1 [1-1] | 1 [1-1] | 1 [1-1] | 1 [1-1] |

**Table 3d: Participants aged 45 to 54 years**

|  | **HaBIDS** | | **Natsal** | |
| --- | --- | --- | --- | --- |
| **Number of sexual partners** | **Men (n=85)** | **Women (n=196)** | **Men (n=730)** | **Women (n=1048)** |
| 0 | 5 (5.9%) | 37 (18.9%) | 120 (16.4%) | 203 (19.4%) |
| 1 | 77 (90.6%) | 148 (75.5%) | 512 (70.1%) | 784 (74.8%) |
| 2 or more | 3 (3.5%) | 11 (5.6%) | 98 (13.4%) | 61 (5.8%) |
| Mean | 1 | 1 | 1 | 1 |
| Median [IQR] | 1 [1-1] | 1 [1-1] | 1 [1-1] | 1 [1-1] |

**Table 3e: Participants aged 55 to 64 years**

|  | **HaBIDS** | | **Natsal** | |
| --- | --- | --- | --- | --- |
| **Number of sexual partners** | **Men (n=105)** | **Women (n=124)** | **Men (n=679)** | **Women (n=957)** |
| 0 | 19 (18.1%) | 37 (29.8%) | 181 (26.7%) | 397 (41.5%) |
| 1 | 81 (77.1%) | 87 (70.2%) | 431 (63.5%) | 540 (56.4%) |
| 2 or more | 5 (4.8%) | 0 (0%) | 67 (9.9%) | 20 (2.1%) |
| Mean | 1 | 1 | 1 | 1 |
| Median [IQR] | 1 [1-1] | 1 [0-1] | 1 [1-1] | 1 [0-1] |

**Table 3f: Participants aged 65 to 74 years**

|  | **HaBIDS** | | **Natsal** | |
| --- | --- | --- | --- | --- |
| **Number of sexual partners** | **Men (n=41)** | **Women (n=49)** | **Men (n=577)** | **Women (n=779)** |
| 0 | 6 (14.6%) | 14 (28.6%) | 259 (44.9%) | 502 (64.4%) |
| 1 | 32 (78.0%) | 33 (67.3%) | 297 (51.5%) | 272 (34.9%) |
| 2 or more | 3 (7.3%) | 2 (4.1%) | 21 (3.6%) | 5 (0.6%) |
| Mean | 2 | 1 | 1 | 0 |
| Median [IQR] | 1 [1-1] | 1 [0-1] | 1 [0-1] | 0 [0-1] |

**Table 4: Frequency of reported number of new opposite-sex sexual partners in the last 12 months by age group and study based on participants who had at least on opposite-sex sexual partner in the last 12 months.**

IQR, interquartile range

**Table 4a: Participants aged 14 to 24 years**

|  | **HaBIDS** | | **Natsal** | |
| --- | --- | --- | --- | --- |
| **Number of sexual partners** | **Men (n=22)** | **Women (n=46)** | **Men (n=1254)** | **Women (n=1642)** |
| 0 | 11 (50.0%) | 15 (32.6%) | 462 (36.8%) | 815 (49.6%) |
| 1 or more | 11 (50.0%) | 31 (67.4%) | 792 (63.2%) | 827 (50.4%) |
| Mean | 1 | 1 | 1 | 1 |
| Median [IQR] | 0 [0-2] | 1 [0-1] | 1 [0-2] | 0 [0-1] |

**Table 4b: Participants aged 25 to 34 years**

|  | **HaBIDS** | | **Natsal** | |
| --- | --- | --- | --- | --- |
| **Number of sexual partners** | **Men (n=45)** | **Women (n=114)** | **Men (n=1336)** | **Women (n=2204)** |
| 0 | 30 (66.7%) | 88 (77.2%) | 886 (66.3%) | 1682 (76.3%) |
| 1 or more | 15 (33.3%) | 26 (22.8%) | 450 (33.7%) | 522 (23.7%) |
| Mean | 1 | 0 | 1 | 0 |
| Median [IQR] | 0 [0-1] | 0 [0-0] | 0 [0-1] | 0 [0-0] |

**Table 4c: Participants aged 35 to 44 years**

|  | **HaBIDS** | | **Natsal** | |
| --- | --- | --- | --- | --- |
| **Number of sexual partners** | **Men (n=67)** | **Women (n=108)** | **Men (n=705)** | **Women (n=1033)** |
| 0 | 51 (76.1%) | 90 (83.3%) | 567 (80.4%) | 861 (83.3%) |
| 1 or more | 16 (23.9%) | 18 (16.7%) | 138 (19.6%) | 172 (16.7%) |
| Mean | 0 | 0 | 0 | 0 |
| Median [IQR] | 0 [0-0] | 0 [0-0] | 0 [0-0] | 0 [0-0] |

**Table 4d: Participants aged 45 to 54 years**

|  | **HaBIDS** | | **Natsal** | |
| --- | --- | --- | --- | --- |
| **Number of sexual partners** | **Men (n=79)** | **Women (n=147)** | **Men (n=618)** | **Women (n=854)** |
| 0 | 72 (91.1%) | 127 (86.4%) | 496 (80.3%) | 727 (85.1%) |
| 1 or more | 7 (8.9%) | 20 (13.6%) | 122 (19.7%) | 127 (14.9%) |
| Mean | 0 | 0 | 0 | 0 |
| Median [IQR] | 0 [0-0] | 0 [0-0] | 0 [0-0] | 0 [0-0] |

**Table 4e: Participants aged 55 to 64 years**

|  | **HaBIDS** | | **Natsal** | |
| --- | --- | --- | --- | --- |
| **Number of sexual partners** | **Men (n=82)** | **Women (n=77)** | **Men (n=503)** | **Women (n=562)** |
| 0 | 75 (91.5%) | 74 (96.1%) | 406 (80.7%) | 508 (90.4%) |
| 1 or more | 7 (8.5%) | 3 (3.9%) | 97 (19.3%) | 54 (9.6%) |
| Mean | 0 | 0 | 0 | 0 |
| Median [IQR] | 0 [0-0] | 0 [0-0] | 0 [0-0] | 0 [0-0] |

**Table 4f: Participants aged 65 to 74 years**

|  | **HaBIDS** | | **Natsal** | |
| --- | --- | --- | --- | --- |
| **Number of sexual partners** | **Men (n=31)** | **Women (n=28)** | **Men (n=322)** | **Women (n=283)** |
| 0 | 30 (96.8%) | 23 (82.1%) | 291 (90.4%) | 267 (94.3%) |
| 1 or more | 1 (3.2%) | 5 (17.9%) | 31 (9.6%) | 16 (5.7%) |
| Mean | 0 | 0 | 0 | 0 |
| Median [IQR] | 0 [0-0] | 0 [0-0] | 0 [0-0] | 0 [0-0] |

**Table 5: Frequency of reported number of lifetime same-sex sexual partners by age group and study**

IQR, interquartile range

**Table 5a: Participants aged 14 to 24 years**

|  | **HaBIDS** | | **SBG** | | **Natsal** | | **NSFG** | |
| --- | --- | --- | --- | --- | --- | --- | --- | --- |
| **Number of sexual partners** | **Men (n=27)** | **Women (n=50)** | **Men (n=126)** | **Women (n=126)** | **Men (n=1725)** | **Women (n=2133)** | **Men (n=4044)** | **Women (n=4321)** |
| 0 | 24 (88.9%) | 45 (90.0%) | 118 (93.7%) | 108 (85.7%) | 1659 (96.2%) | 1972 (92.5%) | 3898 (96.4%) | 3648 (84.4%) |
| 1 | 2 (7.4%) | 2 (4.0%) | 4 (3.2%) | 11 (8.7%) | 21 (1.2%) | 94 (4.4%) | 70 (1.7%) | 372 (8.6%) |
| 2 | 1 (3.7%) | 2 (4.0%) | 1 (0.8%) | 2 (1.6%) | 12 (0.7%) | 25 (1.2%) | 18 (0.4%) | 149 (3.4%) |
| 3-4 | 0 (0%) | 0 (0%) | 0 (0%) | 3 (2.4%) | 10 (0.6%) | 20 (0.9%) | 21 (0.5%) | 95 (2.2%) |
| 5-9 | 0 (0%) | 1 (2.0%) | 1 (0.8%) | 1 (0.8%) | 10 (0.6%) | 15 (0.7%) | 22 (0.5%) | 37 (0.9%) |
| 10 or more | 0 (0%) | 0 (0%) | 2 (1.6%) | 1 (0.8%) | 13 (0.8%) | 7 (0.3%) | 15 (0.4%) | 20 (0.5%) |
| Mean | 0 | 0 | 0 | 0 | 0 | 0 | 0 | 0 |
| Median [IQR] | 0 [0-0] | 0 [0-0] | 0 [0-0] | 0 [0-0] | 0 [0-0] | 0 [0-0] | 0 [0-0] | 0 [0-0] |

**Table 5b: Participants aged 25 to 34 years**

|  | **HaBIDS** | | **SBG** | | **Natsal** | | **NSFG** | |
| --- | --- | --- | --- | --- | --- | --- | --- | --- |
| **Number of sexual partners** | **Men (n=52)** | **Women (n=124)** | **Men (n=156)** | **Women (n=157)** | **Men (n=1518)** | **Women (n=2480)** | **Men (n=3307)** | **Women (n=4369)** |
| 0 | 47 (90.4%) | 103 (83.1%) | 141 (90.4%) | 125 (79.6%) | 1427 (94.0%) | 2263 (91.2%) | 3117 (94.3%) | 3668 (84.0%) |
| 1 | 2 (3.8%) | 12 (9.7%) | 3 (1.9%) | 11 (7.0%) | 28 (1.8%) | 120 (4.8%) | 76 (2.3%) | 356 (8.1%) |
| 2 | 1 (1.9%) | 2 (1.6%) | 1 (0.6%) | 9 (5.7%) | 11 (0.7%) | 44 (1.8%) | 23 (0.7%) | 141 (3.2%) |
| 3-4 | 0 (0%) | 3 (2.4%) | 2 (1.3%) | 6 (3.8%) | 6 (0.4%) | 33 (1.3%) | 22 (0.7%) | 112 (2.6%) |
| 5-9 | 0 (0%) | 2 (1.6%) | 3 (1.9%) | 1 (0.6%) | 11 (0.7%) | 13 (0.5%) | 19 (0.6%) | 63 (1.4%) |
| 10 or more | 2 (3.8%) | 2 (1.6%) | 6 (3.8%) | 5 (3.2%) | 35 (2.3%) | 7 (0.3%) | 50 (1.5%) | 29 (0.7%) |
| Mean | 4 | 0 | 1 | 1 | 2 | 0 | 0 | 0 |
| Median [IQR] | 0 [0-0] | 0 [0-0] | 0 [0-0] | 0 [0-0] | 0 [0-0] | 0 [0-0] | 0 [0-0] | 0 [0-0] |

**Table 5c: Participants aged 35 to 44 years**

|  | **HaBIDS** | | **SBG** | | **Natsal** | | **NSFG** | |
| --- | --- | --- | --- | --- | --- | --- | --- | --- |
| **Number of sexual partners** | **Men (n=78)** | **Women (n=132)** | **Men (n=135)** | **Women (n=157)** | **Men (n=805)** | **Women (n=1209)** | **Men (n=2878)** | **Women (n=3422)** |
| 0 | 71 (91.0%) | 121 (91.7%) | 127 (94.1%) | 136 (86.6%) | 767 (95.3%) | 1123 (92.9%) | 2652 (92.1%) | 2991 (87.4%) |
| 1 | 4 (5.1%) | 6 (4.5%) | 1 (0.7%) | 8 (5.1%) | 11 (1.4%) | 37 (3.1%) | 70 (2.4%) | 221 (6.5%) |
| 2 | 1 (1.3%) | 3 (2.3%) | 1 (0.7%) | 6 (3.8%) | 6 (0.7%) | 18 (1.5%) | 29 (1.0%) | 77 (2.3%) |
| 3-4 | 1 (1.3%) | 1 (0.8%) | 1 (0.7%) | 1 (0.6%) | 7 (0.9%) | 18 (1.5%) | 21 (0.7%) | 73 (2.1%) |
| 5-9 | 1 (1.3%) | 1 (0.8%) | 4 (3.0%) | 3 (1.9%) | 4 (0.5%) | 8 (0.7%) | 26 (0.9%) | 34 (1.0%) |
| 10 or more | 0 (0%) | 0 (0%) | 1 (0.7%) | 3 (1.9%) | 10 (1.2%) | 5 (0.4%) | 80 (2.8%) | 26 (0.8%) |
| Mean | 0 | 0 | 0 | 1 | 2 | 0 | 0 | 0 |
| Median [IQR] | 0 [0-0] | 0 [0-0] | 0 [0-0] | 0 [0-0] | 0 [0-0] | 0 [0-0] | 0 [0-0] | 0 [0-0] |

**Table 5d: Participants aged 45 to 54 years**

|  | **HaBIDS** | | **SBG** | | **Natsal** | |
| --- | --- | --- | --- | --- | --- | --- |
| **Number of sexual partners** | **Men (n=98)** | **Women (n=203)** | **Men (n=184)** | **Women (n=206)** | **Men (n=788)** | **Women (n=1120)** |
| 0 | 89 (90.8%) | 183 (90.1%) | 176 (95.7%) | 185 (89.8%) | 730 (92.6%) | 1040 (92.9%) |
| 1 | 1 (1.0%) | 13 (6.4%) | 2 (1.1%) | 8 (3.9%) | 21 (2.7%) | 41 (3.7%) |
| 2 | 2 (2.0%) | 1 (0.5%) | 1 (0.5%) | 3 (1.5%) | 8 (1.0%) | 13 (1.2%) |
| 3-4 | 2 (2.0%) | 4 (2.0%) | 1 (0.5%) | 5 (2.4%) | 6 (0.8%) | 17 (1.5%) |
| 5-9 | 1 (1.0%) | 2 (1.0%) | 2 (1.1%) | 2 (1.0%) | 8 (1.0%) | 5 (0.4%) |
| 10 or more | 3 (3.1%) | 0 (0%) | 2 (1.1%) | 3 (1.5%) | 15 (1.9%) | 4 (0.4%) |
| Mean | 1 | 0 | 0 | 0 | 2 | 0 |
| Median [IQR] | 0 [0-0] | 0 [0-0] | 0 [0-0] | 0 [0-0] | 0 [0-0] | 0 [0-0] |

**Table 5e: Participants aged 55 to 64 years**

|  | **HaBIDS** | | **SBG** | | **Natsal** | |
| --- | --- | --- | --- | --- | --- | --- |
| **Number of sexual partners** | **Men (n=115)** | **Women (n=134)** | **Men (n=157)** | **Women (n=197)** | **Men (n=768)** | **Women (n=1028)** |
| 0 | 104 (90.4%) | 116 (86.6%) | 149 (94.9%) | 186 (94.4%) | 711 (92.6%) | 1001 (97.4%) |
| 1 | 4 (3.5%) | 6 (4.5%) | 3 (1.9%) | 5 (2.5%) | 18 (2.3%) | 15 (1.5%) |
| 2 | 0 (0%) | 4 (3.0%) | 1 (0.6%) | 3 (1.5%) | 8 (1.0%) | 7 (0.7%) |
| 3-4 | 3 (2.6%) | 2 (1.5%) | 3 (1.9%) | 1 (0.5%) | 10 (1.3%) | 3 (0.3%) |
| 5-9 | 3 (2.6%) | 6 (4.5%) | 0 (0%) | 0 (0%) | 8 (1.0%) | 2 (0.2%) |
| 10 or more | 1 (0.9%) | 0 (0%) | 1 (0.6%) | 2 (1.0%) | 13 (1.7%) | 0 (0%) |
| Mean | 0 | 0 | 4 | 0 | 1 | 0 |
| Median [IQR] | 0 [0-0] | 0 [0-0] | 0 [0-0] | 0 [0-0] | 0 [0-0] | 0 [0-0] |

**Table 5f: Participants aged 65 to 74 years**

|  | **HaBIDS** | | **SBG** | | **Natsal** | |
| --- | --- | --- | --- | --- | --- | --- |
| **Number of sexual partners** | **Men (n=41)** | **Women (n=50)** | **Men (n=139)** | **Women (n=111)** | **Men (n=657)** | **Women (n=871)** |
| 0 | 38 (92.7%) | 44 (88.0%) | 134 (96.4%) | 105 (94.6%) | 638 (97.1%) | 863 (99.1%) |
| 1 | 2 (4.9%) | 5 (10.0%) | 1 (0.7%) | 1 (0.9%) | 7 (1.1%) | 4 (0.5%) |
| 2 | 1 (2.4%) | 0 (0%) | 1 (0.7%) | 3 (2.7%) | 4 (0.6%) | 0 (0%) |
| 3-4 | 0 (0%) | 0 (0%) | 1 (0.7%) | 0 (0%) | 3 (0.5%) | 1 (0.1%) |
| 5-9 | 0 (0%) | 1 (2.0%) | 0 (0%) | 1 (0.9%) | 2 (0.3%) | 1 (0.1%) |
| 10 or more | 0 (0%) | 0 (0%) | 2 (1.4%) | 1 (0.9%) | 3 (0.5%) | 2 (0.2%) |
| Mean | 0 | 0 | 0 | 0 | 1 | 0 |
| Median [IQR] | 0 [0-0] | 0 [0-0] | 0 [0-0] | 0 [0-0] | 0 [0-0] | 0 [0-0] |

**Table 6: Frequency of reported number of same-sex sexual partners in the last 12 months by age group and study based on participants who had at least one lifetime same-sex sexual partner.**

IQR, interquartile range.

**Table 6a: Participants aged 14 to 24 years**

|  | **HaBIDS** | | **Natsal** | | **NSFG** | |
| --- | --- | --- | --- | --- | --- | --- |
| **Number of sexual partners** | **Men (n=3)** | **Women (n=5)** | **Men (n=65)** | **Women (n=161)** | **Men (n=144)** | **Women (n=672)** |
| 0 | 2 (66.7%) | 1 (20.0%) | 20 (30.8%) | 83 (51.6%) | 68 (47.2%) | 363 (54.0%) |
| 1 | 0 (0%) | 2 (40.0%) | 22 (33.8%) | 59 (36.6%) | 37 (25.7%) | 221 (32.9%) |
| 2 or more | 1 (33.3%) | 2 (40.0%) | 23 (35.4%) | 19 (11.8%) | 39 (27.1%) | 88 (13.1%) |
| Mean | 0 | 0 | 0 | 0 | 0 | 0 |
| Median [IQR] | 0 [0-0] | 0 [0-0] | 0 [0-0] | 0 [0-0] | 0 [0-0] | 0 [0-0] |

**Table 6b: Participants aged 25 to 34 years**

|  | **HaBIDS** | | **Natsal** | | **NSFG** | |
| --- | --- | --- | --- | --- | --- | --- |
| **Number of sexual partners** | **Men (n=5)** | **Women (n=19)** | **Men (n=91)** | **Women (n=216)** | **Men (n=190)** | **Women (n=701)** |
| 0 | 2 (40.0%) | 11 (57.9%) | 41 (45.1%) | 150 (69.4%) | 97 (51.1%) | 505 (72.0%) |
| 1 | 1 (20.0%) | 7 (36.8%) | 24 (26.4%) | 56 (25.9%) | 44 (23.2%) | 148 (21.1%) |
| 2 or more | 2 (40.0%) | 1 (5.3%) | 26 (28.6%) | 10 (4.6%) | 49 (25.8%) | 48 (6.8%) |
| Mean | 0 | 0 | 0 | 0 | 0 | 0 |
| Median [IQR] | 0 [0-0] | 0 [0-0] | 0 [0-0] | 0 [0-0] | 0 [0-0] | 0 [0-0] |

**Table 6c: Participants aged 35 to 44 years**

|  | **HaBIDS** | | **Natsal** | | **NSFG** | |
| --- | --- | --- | --- | --- | --- | --- |
| **Number of sexual partners** | **Men (n=6)** | **Women (n=10)** | **Men (n=38)** | **Women (n=85)** | **Men (n=226)** | **Women (n=431)** |
| 0 | 5 (83.3%) | 7 (70.0%) | 21 (55.3%) | 51 (60.0%) | 131 (58.0%) | 323 (74.9%) |
| 1 | 1 (16.7%) | 3 (30.0%) | 8 (21.1%) | 28 (32.9%) | 39 (17.3%) | 89 (20.6%) |
| 2 or more | 0 (0%) | 0 (0%) | 9 (23.7%) | 6 (7.1%) | 56 (24.8%) | 19 (4.4%) |
| Mean | 0 | 0 | 0 | 0 | 0 | 0 |
| Median [IQR] | 0 [0-0] | 0 [0-0] | 0 [0-0] | 0 [0-0] | 0 [0-0] | 0 [0-0] |

**Table 6d: Participants aged 45 to 54 years**

|  | **HaBIDS** | | **Natsal** | |
| --- | --- | --- | --- | --- |
| **Number of sexual partners** | **Men (n=9)** | **Women (n=20)** | **Men (n=57)** | **Women (n=79)** |
| 0 | 3 (33.3%) | 10 (50.0%) | 33 (57.9%) | 51 (64.6%) |
| 1 | 6 (66.7%) | 6 (30.0%) | 12 (21.1%) | 22 (27.8%) |
| 2 or more | 0 (0%) | 4 (20.0%) | 12 (21.1%) | 6 (7.6%) |
| Mean | 0 | 0 | 0 | 0 |
| Median [IQR] | 0 [0-0] | 0 [0-0] | 0 [0-0] | 0 [0-0] |

**Table 6e: Participants aged 55 to 64 years**

|  | **HaBIDS** | | **Natsal** | |
| --- | --- | --- | --- | --- |
| **Number of sexual partners** | **Men (n=10)** | **Women (n=15)** | **Men (n=57)** | **Women (n=27)** |
| 0 | 4 (40.0%) | 6 (40.0%) | 41 (71.9%) | 21 (77.8%) |
| 1 | 6 (60.0%) | 9 (60.0%) | 8 (14.0%) | 6 (22.2%) |
| 2 or more | 0 (0%) | 0 (0%) | 8 (14.0%) | 0 (0%) |
| Mean | 0 | 0 | 0 | 0 |
| Median [IQR] | 0 [0-0] | 0 [0-0] | 0 [0-0] | 0 [0-0] |

**Table 6f: Participants aged 65 to 74 years**

|  | **HaBIDS** | | **Natsal** | |
| --- | --- | --- | --- | --- |
| **Number of sexual partners** | **Men (n=3)** | **Women (n=5)** | **Men (n=19)** | **Women (n=8)** |
| 0 | 2 (66.7%) | 1 (20.0%) | 14 (73.7%) | 7 (87.5%) |
| 1 | 1 (33.3%) | 4 (80.0%) | 3 (15.8%) | 1 (12.5%) |
| 2 or more | 0 (0%) | 0 (0%) | 2 (10.5%) | 0 (0%) |
| Mean | 0 | 0 | 0 | 0 |
| Median [IQR] | 0 [0-0] | 0 [0-0] | 0 [0-0] | 0 [0-0] |

**Table 7: Frequency of reported number of new same-sex sexual partners in the last 12 months by age group and study based on participants who had at least one same-sex sexual partner in the last 12 months.**

IQR, interquartile range

**Table 7a: Participants aged 14 to 24 years**

|  | **HaBIDS** | | **Natsal** | |
| --- | --- | --- | --- | --- |
| **Number of sexual partners** | **Men (n=1)** | **Women (n=4)** | **Men (n=45)** | **Women (n=78)** |
| 0 | 0 (0%) | 1 (25.0%) | 10 (22.2%) | 34 (43.6%) |
| 1 or more | 1 (100%) | 3 (75.0%) | 35 (77.8%) | 44 (56.4%) |
| Mean | 0 | 0 | 0 | 0 |
| Median [IQR] | 0 [0-0] | 0 [0-0] | 0 [0-0] | 0 [0-0] |

**Table 7b: Participants aged 25 to 34 years**

|  | **HaBIDS** | | **Natsal** | |
| --- | --- | --- | --- | --- |
| **Number of sexual partners** | **Men (n=3)** | **Women (n=7)** | **Men (n=49)** | **Women (n=67)** |
| 0 | 0 (0%) | 4 (57.1%) | 21 (42.9%) | 46 (68.7%) |
| 1 or more | 3 (100%) | 3 (42.9%) | 28 (57.1%) | 21 (31.3%) |
| Mean | 0 | 0 | 0 | 0 |
| Median [IQR] | 0 [0-0] | 0 [0-0] | 0 [0-0] | 0 [0-0] |

**Table 7c: Participants aged 35 to 44 years**

|  | **HaBIDS** | | **Natsal** | |
| --- | --- | --- | --- | --- |
| **Number of sexual partners** | **Men (n=1)** | **Women (n=2)** | **Men (n=17)** | **Women (n=34)** |
| 0 | 1 (100%) | 2 (100%) | 9 (52.9%) | 21 (61.8%) |
| 1 or more | 0 (0%) | 0 (0%) | 8 (47.1%) | 13 (38.2%) |
| Mean | 0 | 0 | 0 | 0 |
| Median [IQR] | 0 [0-0] | 0 [0-0] | 0 [0-0] | 0 [0-0] |

**Table 7d: Participants aged 45 to 54 years**

|  | **HaBIDS** | | **Natsal** | |
| --- | --- | --- | --- | --- |
| **Number of sexual partners** | **Men (n=6)** | **Women (n=9)** | **Men (n=24)** | **Women (n=28)** |
| 0 | 5 (83.3%) | 5 (55.6%) | 15 (62.5%) | 19 (67.9%) |
| 1 or more | 1 (16.7%) | 4 (44.4%) | 9 (37.5%) | 9 (32.1%) |
| Mean | 0 | 0 | 0 | 0 |
| Median [IQR] | 0 [0-0] | 0 [0-0] | 0 [0-0] | 0 [0-0] |

**Table 7e: Participants aged 55 to 64 years**

|  | **HaBIDS** | | **Natsal** | |
| --- | --- | --- | --- | --- |
| **Number of sexual partners** | **Men (n=5)** | **Women (n=5)** | **Men (n=17)** | **Women (n=6)** |
| 0 | 4 (80.0%) | 4 (80.0%) | 7 (41.2%) | 5 (83.3%) |
| 1 or more | 1 (20.0%) | 1 (20.0%) | 10 (58.8%) | 1 (16.7%) |
| Mean | 0 | 0 | 0 | 0 |
| Median [IQR] | 0 [0-0] | 0 [0-0] | 0 [0-0] | 0 [0-0] |

**Table 7f: Participants aged 65 to 74 years**

|  | **HaBIDS** | | **Natsal** | |
| --- | --- | --- | --- | --- |
| **Number of sexual partners** | **Men (n=1)** | **Women (n=1)** | **Men (n=6)** | **Women (n=1)** |
| 0 | 1 (100%) | 1 (100%) | 3 (50.0%) | 1 (100%) |
| 1 or more | 0 (0%) | 0 (0%) | 3 (50.0%) | 0 (0%) |
| Mean | 0 | 0 | 0 | 0 |
| Median [IQR] | 0 [0-0] | 0 [0-0] | 0 [0-0] | 0 [0-0] |

**Table 8: Frequency of reported number of participants with both-sex experience by study.**

The number of sexual partners in the last 12 months is based on participants who had at least one lifetime opposite- and one same-sex sexual partner. Number of new sexual partners in the last 12 months is based on participants who had at least one opposite- and one same-sex sexual partner in the last 12 months.

IQR, interquartile range

**Table 8a: Participants with both-sex experience in the entire life**

|  | **HaBIDS** | | **SBG** | | **Natsal** | | **NSFG** | |
| --- | --- | --- | --- | --- | --- | --- | --- | --- |
| **Number of sexual partners** | **Men (n=31)** | **Women (n=78)** | **Men (n=31)** | **Women (n=88)** | **Men (n=247)** | **Women (n=536)** | **Men (n=447)** | **Women (n=1705)** |
| 0 | 0 (0%) | 0 (0%) | 0 (0%) | 0 (0%) | 0 (0%) | 0 (0%) | 0 (0%) | 0 (0%) |
| 1 | 0 (0%) | 0 (0%) | 0 (0%) | 0 (0%) | 0 (0%) | 0 (0%) | 0 (0%) | 0 (0%) |
| 2 | 4 (12.9%) | 16 (20.5%) | 4 (12.9%) | 4 (4.5%) | 6 (2.4%) | 15 (2.8%) | 23 (5.1%) | 67 (3.9%) |
| 3-4 | 6 (19.4%) | 11 (14.1%) | 0 (0%) | 9 (10.2%) | 22 (8.9%) | 33 (6.2%) | 37 (8.3%) | 154 (9.0%) |
| 5-9 | 11 (35.5%) | 25 (32.1%) | 8 (25.8%) | 24 (27.3%) | 70 (28.3%) | 143 (26.7%) | 110 (24.6%) | 523 (30.7%) |
| 10 or more | 10 (32.3%) | 26 (33.3%) | 19 (61.3%) | 51 (58.0%) | 149 (60.3%) | 345 (64.4%) | 277 (62.0%) | 961 (56.4%) |
| Mean | 16 | 10 | 65 | 17 | 55 | 21 | 16 | 16 |
| Median [IQR] | 7 [4-12] | 7 [4-11] | 13 [6-49] | 11 [7-17] | 15 [7-31] | 12 [7-21] | 12 [6-20] | 11 [6-20] |

**Table 8b: Participants with both-sex experience in the last 12 months**

|  | **HaBIDS** | | **Natsal** | | **NSFG** | |
| --- | --- | --- | --- | --- | --- | --- |
| **Number of sexual partners** | **Men (n=29)** | **Women (n=71)** | **Men (n=247)** | **Women (n=534)** | **Men (n=446)** | **Women (n=1705)** |
| 0 | 1 (3.4%) | 8 (11.3%) | 30 (12.1%) | 41 (7.7%) | 53 (11.9%) | 83 (4.9%) |
| 1 | 14 (48.3%) | 30 (42.3%) | 115 (46.6%) | 272 (50.9%) | 198 (44.4%) | 844 (49.5%) |
| 2 or more | 14 (48.3%) | 33 (46.5%) | 102 (41.3%) | 221 (41.4%) | 195 (43.7%) | 778 (45.6%) |
| Mean | 2 | 2 | 3 | 3 | 2 | 2 |
| Median [IQR] | 1 [1-2] | 2 [1-2] | 1 [1-3] | 1 [1-2] | 1 [1-2] | 1 [1-2] |

**Table 8c: Participants with new both-sex experience in the last 12 months**

|  | **HaBIDS** | | **Natsal** | |
| --- | --- | --- | --- | --- |
| **Number of sexual partners** | **Men (n=28)** | **Women (n=52)** | **Men (n=221)** | **Women (n=497)** |
| 0 | 18 (64.3%) | 32 (61.5%) | 114 (51.6%) | 277 (55.7%) |
| 1 or more | 10 (35.7%) | 20 (38.5%) | 107 (48.4%) | 220 (44.3%) |
| Mean | 1 | 1 | 2 | 1 |
| Median [IQR] | 0 [0-1] | 0 [0-1] | 0 [0-2] | 0 [0-1] |

**Figure 1:** Comparison of age trend in the frequency of reported number of sexual partners within studies. Percentage of lifetime opposite-sex sexual partners across all studies by sex and age for (A) all participants (B) male participants (C) female participants


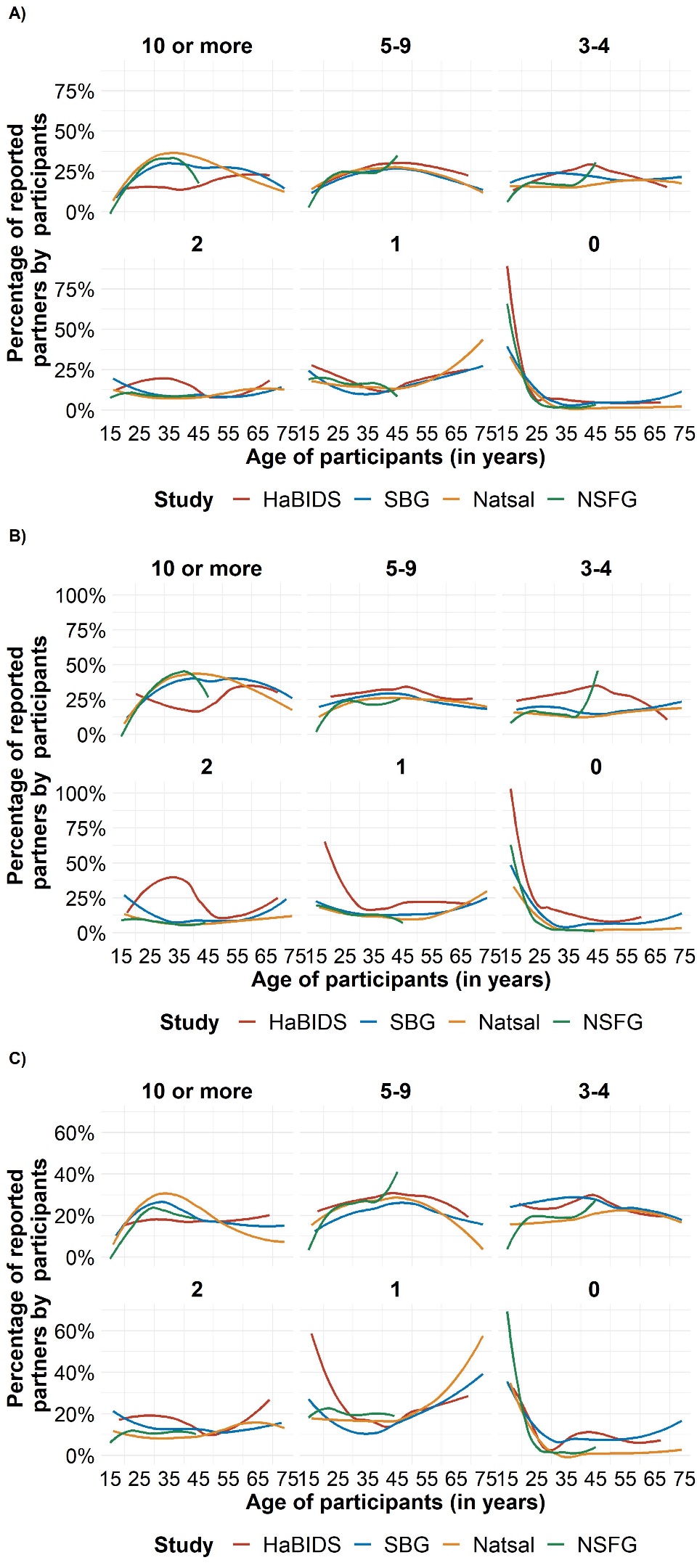


**Figure 2**: Percentage of opposite-sex sexual partners across all studies by sex and age in the last 12 months based on participants who had at least one lifetime opposite-sex sexual partner for (A) all participants (B) male participants (C) female participants


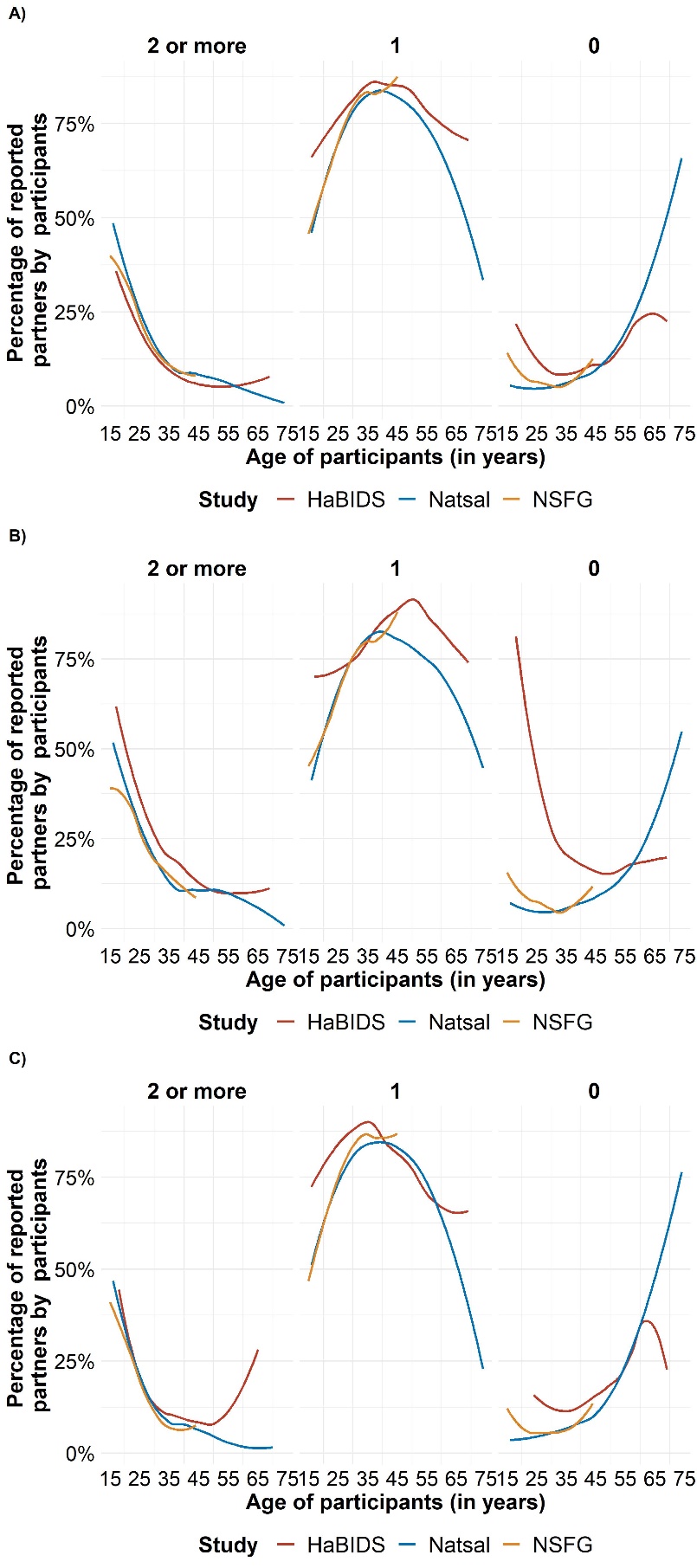


**Figure 3**: Percentage of new opposite-sex sexual partners across all studies by sex and age in the last 12 months is based on participants who had at least one opposite-sex sexual partner in the last 12 months for (A) all participants (B) male participants (C) female participants


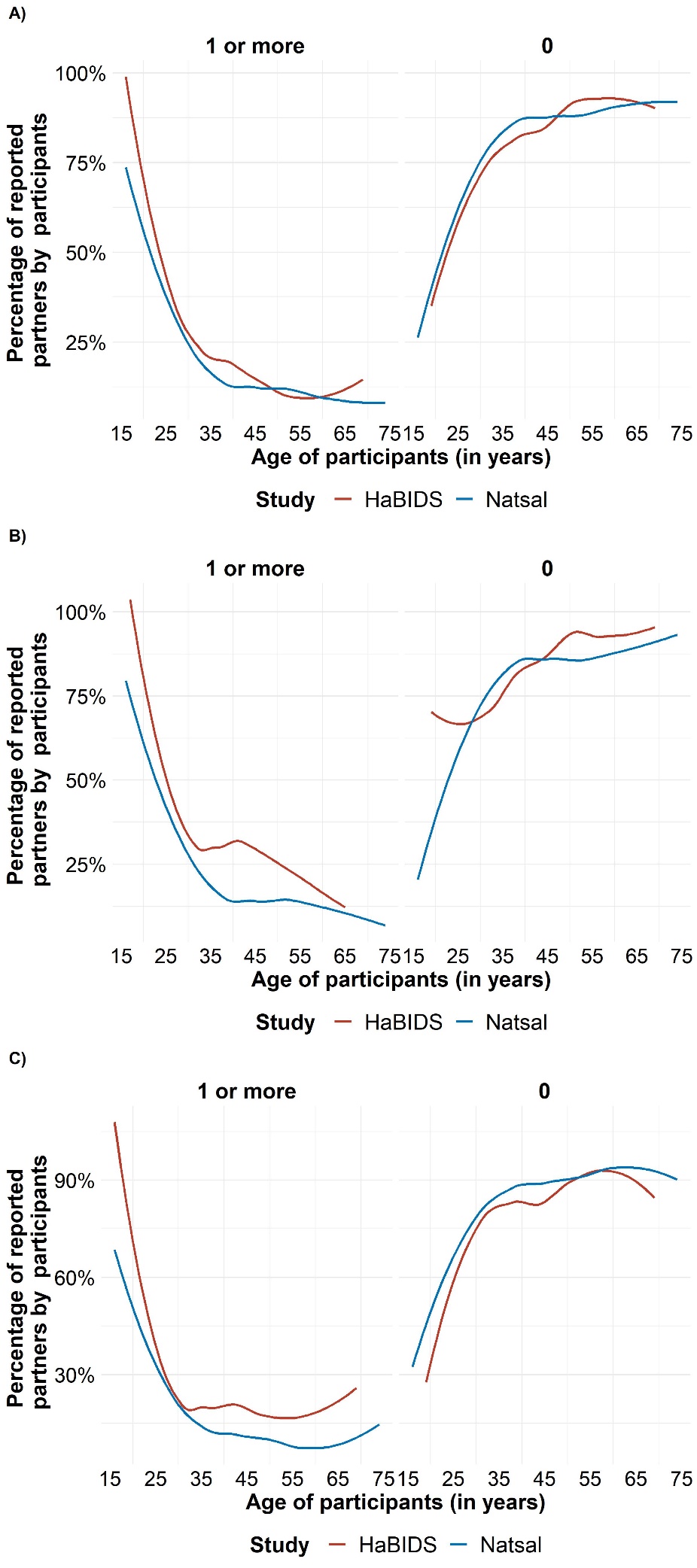


**Figure 4**: Comparison between studies on the number of opposite-sex sexual partners by age (A) in the participants’ entire life (B) in the last 12 months (based on participants who had at least one lifetime opposite-sex sexual partner) and (C) new opposite-sex sexual partners in the last 12 months (based on participants who had at least one opposite-sex sexual partner in the last 12 months)


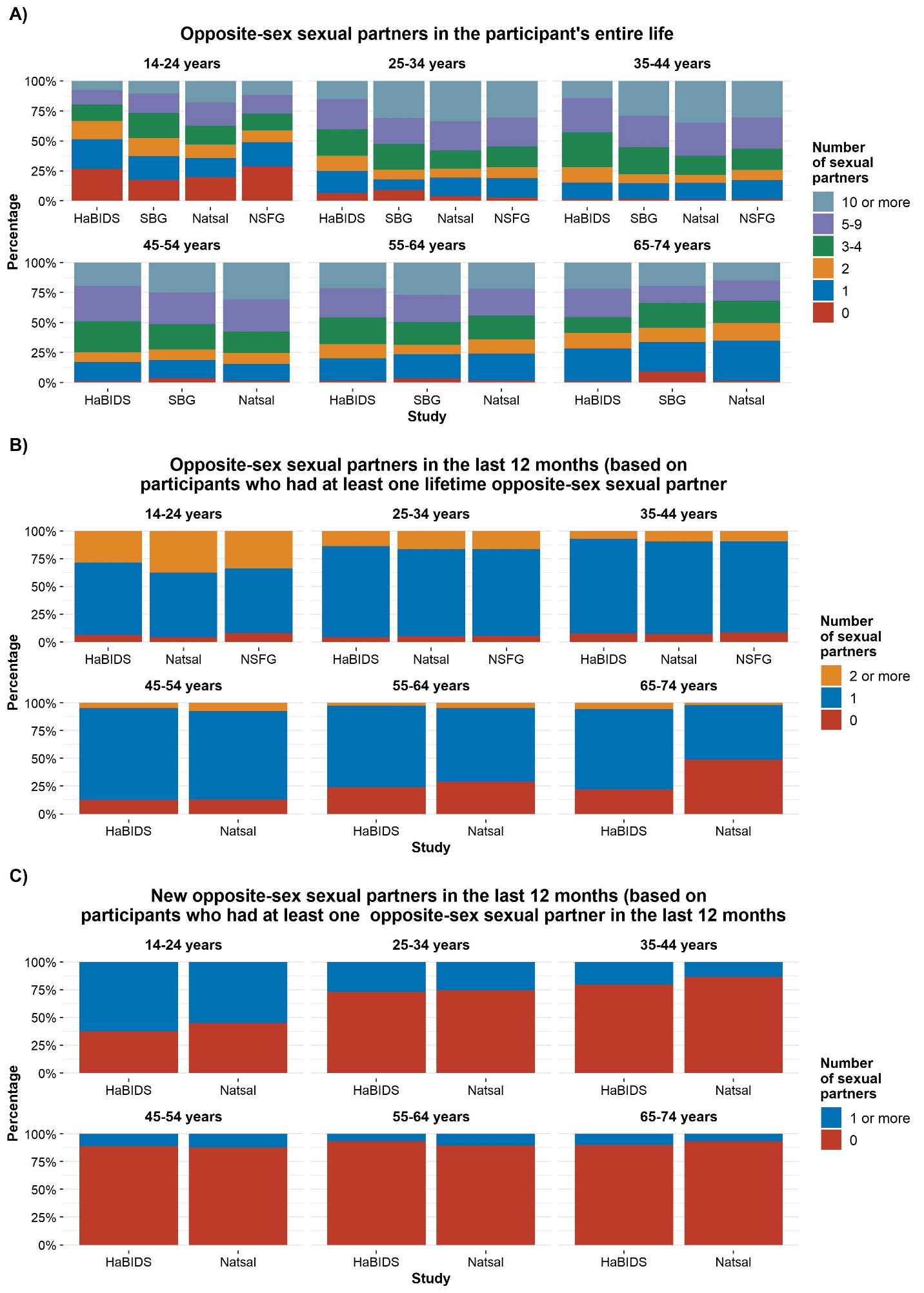


.

**Figure 5**: Percentage of lifetime same-sex sexual partners across all studies by sex and age


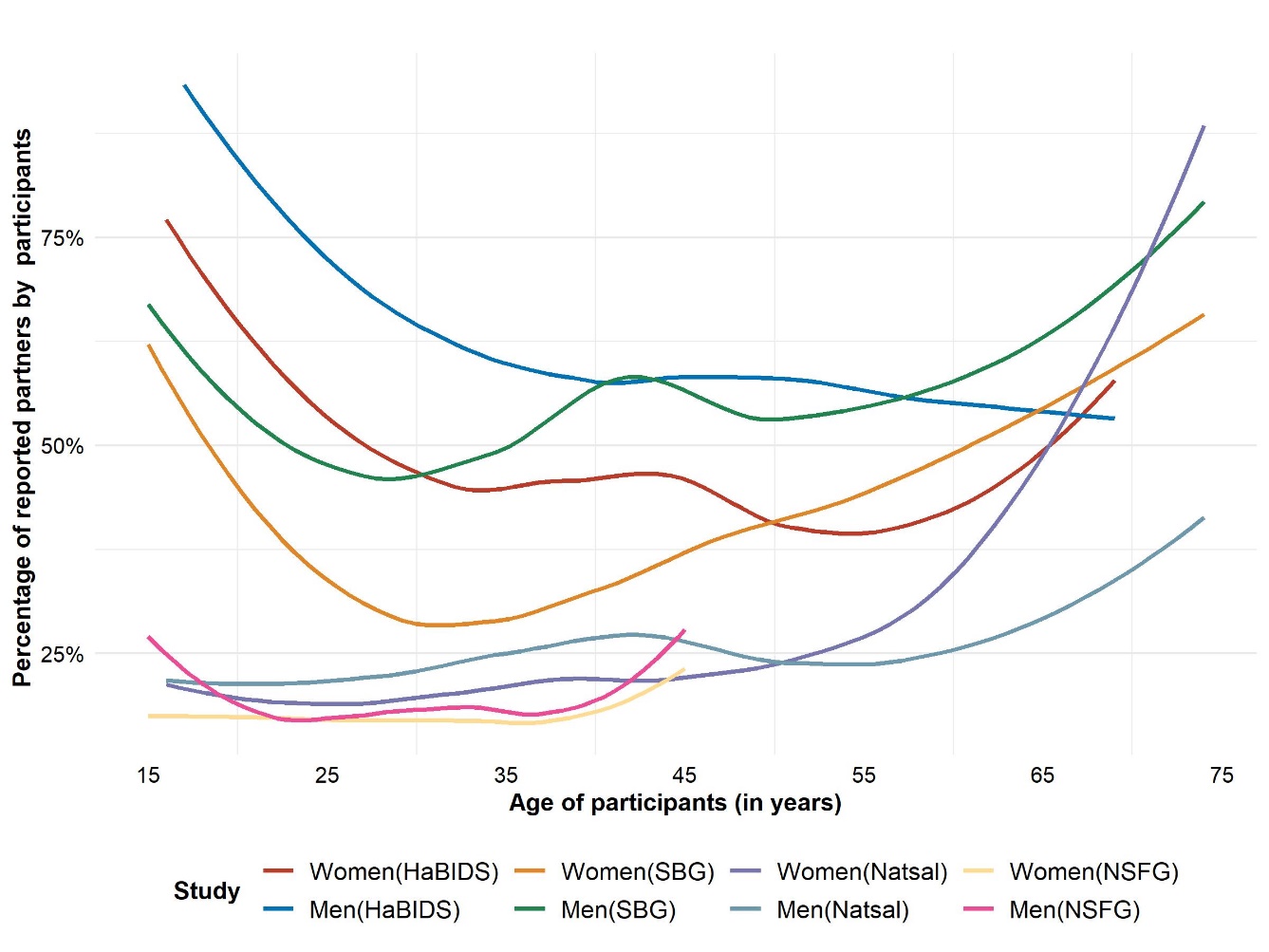


**English translation of the questionnaire on** **Sexual contact patterns**

*(German questionnaire translated into English; English versions not validated for use in surveys)*

▼ indicates that the question is displayed only if the respective condition is met.

[ ] Variable names are written in square brackets and are not visible to the participants.

In the following, we ask for sexual contacts, that is, those with whom sexual intercourse has taken place. There are diseases that can be transmitted via sexual intercourse. The transmission takes place only sometimes and not everyone is affected. We ask these questions to understand the possible spread of these pathogens. This is not about whether someone was or is ill. It is also very important to get answers from people who don’t have sexually transmitted diseases. Again, we would like to stress at this point that your answers will remain anonymous and no individual responses will be visible. The answer to the questions is, of course, voluntary.

[keine_Antwort] If you don’t want to complete this section of the survey, please let us know.

In the course of the survey you have also the option "I don’t want to answer".

| □ | I don’t want to complete this section of the survey. |
| --- | --- |

[sex1] How old were you when you first had sexual intercourse?

If you are not sure how old you were, please give an estimate.

▼ keine_Antwort_!= “Yes”

| □ | I don’t want to answer. |
| --- | --- |
| □ | I have not had any sexual contact yet. |
| □ | My age in years was then: |

[sex2] Do you currently have a sexual partnership?

Sexual partnership means: a partnership in which sexual intercourse takes place.

▼ (keine_Antwort_!= “Yes”) & (sex1 != “I have not had any sexual contact yet.”)

| □ | Yes |
| --- | --- |
| □ | No |
| □ | I don’t want to answer. |

[sex2_dauer] Do you think that this sexual partnership will continue in 3 months?

▼ sex2 == “Yes”

| □ | Yes |
| --- | --- |
| □ | No |
| □ | Don‘t know |
| □ | I don’t want to answer. |

## Contacts with persons of the opposite-sex

[sex3] If you think about your whole life, with how many people of the opposite-sex have you had sexual intercourse so far?

|  | *enter your answer here* |
| --- | --- |
| □ | I don’t want to answer. |

[sex4] If you think about the last 12 months (since June 1, 2014), with how many people of the opposite-sex did you have sexual intercourse?

▼ sex3_1_anz >= “1”

|  | *enter your answer here* |
| --- | --- |
| □ | I don’t want to answer. |

[sex4neu] How many of these {sex4_1_anz} people you have had sexual intercourse with over the past 12 months have been new partners with whom you have had sexual intercourse for the first time?

▼ sex4_1_anz >= “1”

|  | *enter your answer here* |
| --- | --- |
| □ | I don’t want to answer. |

## Contacts with persons of the same-sex

[sex5] If you think about your entire life, with how many people of the same-sex have you had sexual intercourse so far?

|  | *enter your answer here* |
| --- | --- |
| □ | I don’t want to answer. |

[sex6] If you think about the last 12 months (since June 1, 2014), with how many people of the same-sex did you have sexual intercourse?

▼ sex5_1_anz >= “1”

|  | *enter your answer here* |
| --- | --- |
| □ | I don’t want to answer. |

[sex6neu] How many of these {sex6_1_anz} people you have had sexual intercourse with over the last 12 months have been new partners with whom you had sexual intercourse for the first time?

▼ sex6_1_anz >= “1”

|  | *enter your answer here* |
| --- | --- |
| □ | I don’t want to answer. |

## Age of contact persons

[sex7_99] How old were the last partners with whom you had sexual intercourse?

Please refer only to your sexual partners during the last 12 months (since June 1, 2014).

Please also provide details of how often you / your partner used condoms when having sexual intercourse.

| □ | I don’t want to answer. |
| --- | --- |

If you don’t know the exact age, please give your best estimate of age.

|  | Age | Gender | | Frequency of condom use | | |
| --- | --- | --- | --- | --- | --- | --- |
|  |  | Male | Female | Always  (for every sexual intercourse) | Sometimes yes,  sometimes no | Never |
| last or current partner |  | □ | □ | □ | □ | □ |
| Penultimate partner |  | □ | □ | □ | □ | □ |
| Pre-penultimate partner |  | □ | □ | □ | □ | □ |

## Contacts of the partner

{if (sex2 == “Yes”, “The following questions pertain to your current partner(s). "," The following questions refer to your last sexual partnership. ")}

[sex9] Did your partners have sexual intercourse with other people in the last 12 months (since June 1, 2014)?

| □ | Yes (I know exactly) |
| --- | --- |
| □ | Yes (I suppose, but I don’t know) |
| □ | No (I know exactly) |
| □ | I don’t know it |
| □ | I don’t want to answer. |

[sex9_anz] Do you know how many sexual partners your partners have had in the last 12 months (excluding yourself)?

▼ sex9 == “Yes

| □ | No, I don’t know that. |
| --- | --- |
| □ | I don’t want to answer. |
| □ | Yes, number of partners: |

[sex10] Do you know how many sexual partners your partners have had in your life (excluding yourself)?

| □ | No, I don’t know that. |
| --- | --- |
| □ | I don’t want to answer. |
| □ | Yes, number of partners: |

[sex11] How often did your partner use a condom in the past partnerships in his / her life?

| □ | Always (for every sexual intercourse) |
| --- | --- |
| □ | Sometimes yes, sometimes no |
| □ | Never |
| □ | Do not know |
| □ | I do not want to answer. |
